# Local Structure and Magnetism of Fe_2_O_3_ Maghemite Nanocrystals: The Role of Crystal Dimension

**DOI:** 10.3390/nano10050867

**Published:** 2020-04-30

**Authors:** Mauro Coduri, Paolo Masala, Lucia Del Bianco, Federico Spizzo, Davide Ceresoli, Carlo Castellano, Serena Cappelli, Cesare Oliva, Stefano Checchia, Mattia Allieta, Dorothee-Vinga Szabo, Sabine Schlabach, Michael Hagelstein, Claudio Ferrero, Marco Scavini

**Affiliations:** 1 Department of Chemistry, University of Pavia, viale Taramelli 16, 27100 Pavia, Italy; 2 Department of Chemistry, University of Milan, via Golgi 19, 20131 Milano, Italy; masala1983@libero.it (P.M.); carlo.castellano@unimi.it (C.C.); serena.cappelli@unimi.it (S.C.); cesare.oliva@unimi.it (C.O.); mattia.allieta@gmail.com (M.A.); 3 Department of Physics and Earth Sciences, University of Ferrara, via Saragat 1, 44122 Ferrara, Italy; lucia.delbianco@unife.it (L.D.B.); federico.spizzo@unife.it (F.S.); 4 National Research Council of Italy, Institute of Chemical Science and Technology (CNR-SCITEC), 20133 Milano, Italy; davide.ceresoli@cnr.it; 5 MAX IV Laboratory, Lund University, 22100 Lund, Sweden; stefano.checchia@maxiv.lu.se; 6 Karlsruhe Institute of Technology, Institute for Applied Materials (IAM) and Karlsruhe Nano Micro Facility (KNMF), Hermann-von-Helmholtz-Platz 1, 76344 Eggenstein-Leopoldshafen, Germany; dorothee.szabo@kit.edu (D.-V.S.); sabine.schlabach@kit.edu (S.S.); 7 Karlsruhe Institute of Technology, Institute for Beam Physics and Technology (IBPT), Hermann-von-Helmholtz-Platz 1, 76344 Eggenstein-Leopoldshafen, Germany; michael.hagelstein@kit.edu; 8 European Synchrotron Radiation Facility, 38000 Grenoble, France; ferrero@esrf.fr

**Keywords:** maghemite, pair distribution function, disordered magnetism, X-ray absorption spectroscopy, molecular dynamics, finite size effects

## Abstract

Here we report on the impact of reducing the crystalline size on the structural and magnetic properties of γ-Fe_2_O_3_ maghemite nanoparticles. A set of polycrystalline specimens with crystallite size ranging from ~2 to ~50 nm was obtained combining microwave plasma synthesis and commercial samples. Crystallite size was derived by electron microscopy and synchrotron powder diffraction, which was used also to investigate the crystallographic structure. The local atomic structure was inquired combining pair distribution function (PDF) and X-ray absorption spectroscopy (XAS). PDF revealed that reducing the crystal dimension induces the depletion of the amount of Fe tetrahedral sites. XAS confirmed significant bond distance expansion and a loose Fe-Fe connectivity between octahedral and tetrahedral sites. Molecular dynamics revealed important surface effects, whose implementation in PDF reproduces the first shells of experimental curves. The structural disorder affects the magnetic properties more and more with decreasing the nanoparticle size. In particular, the saturation magnetization reduces, revealing a spin canting effect. Moreover, a large effective magnetic anisotropy is measured at low temperature together with an exchange bias effect, a behavior that we related to the existence of a highly disordered glassy magnetic phase.

## 1. Introduction

Even before the advent of “nanoscale science” and “nanotechnology” as recognized fields of science and engineering, small particles, colloids, and high-surface-area materials have been critical in applications ranging from catalysis to waste management [1,2]. When dealing with very small nanoparticles, surface effects become predominant and the very notions of “phase” and “surface” become murky. When it comes to magnetic materials, new fascinating properties can be achieved by reducing the size of the nanoparticles with potential applications to storage devices and medical imaging [3,4]. Hence the study of the interrelation between microstructure and magnetism is very appealing. In addition, very different magnetic properties have been observed with materials having similar grain sizes but produced by different synthesis routes [5,6,7,8] and, therefore, having different microstructures. As an example, many research activities are currently focused in understanding and tailoring the properties of magnetic nanoparticles for nanomedicine applications [9,10,11,12,13,14]. It has been reported often that the magnetic parameters of spinel iron oxides, e.g., saturation magnetization and coercivity, are affected by the size reduction at the nanometer scale, also depending on the synthesis method [6,15,16]. Many studies are devoted to the control and the understanding of the synthesis to produce nanoparticles with tailored characteristics, [6,7,17,18,19,20,21,22], while more limited are the correlations with their crystal structure and microstructure [19,23,24,25]. Whether this can origin from the formation of complex architectures [7,19,26,27], a general limitation is given by the complexity of resolving the crystal structures of γ-Fe_2_O_3_ maghemite and Fe_3_O_4_ magnetite, since both phases have a cubic structure with very similar lattice parameter, which are more difficult to resolve when the crystal size is small.

Recently, advanced crystallographic investigations in the field of Fe oxides have been dedicated mostly to the understanding of the structure modifications across the Verwey transition in magnetite [28,29,30] and to exploring new higher order phases such as Fe_4_O_5_ [31,32], their XFe_3_O_5_ modifications [33,34,35,36] and recently even Fe_5_O_6_ [37] and other unconventional stoichiometries.

However, despite the wide literature available on magnetic nanoparticles, the crystallographic investigations aimed at unveiling the disorder at the atomic scale are scarce and generally dedicated to magnetite or peculiar shaped composites. Among others, Salazar et al. [27] and Frison et al. [38] provided a systematic investigation of magnetite/maghemite core-shell nanocrystals, while Petkov et al. [39] compared spheres and tetrapods with reference to grain growth and magnetic properties. Only recently, the atomic scale mechanism of formation of maghemite nanoparticles was observed by in-situ total scattering [40].

Here, we investigate a set of γ-Fe_2_O_3_ specimens with progressive crystal size dimension, from a couple to tens of nanometers, with the goal of exploring the impact of small crystal size onto the atomic structure and its consequences on the magnetic properties. To this purpose, we combined different synchrotron techniques, such as high-resolution powder diffraction, pair distribution function PDF, X-ray absorption near edge structure (XANES) and extended X-ray absorption fine structure (EXAFS), comparing results using molecular dynamics. The impact of crystal size on the magnetothermal properties is evaluated via superconducting quantum interference device (SQUID) and electron magnetic resonance (EMR) spectroscopy.

## 2. Materials and Methods

Two specimens of Fe_2_O_3_ nanoparticles have been synthesized by microwave plasma synthesis, a non-equilibrium gas phase method, which is able to generate nanoparticles with sizes around and below 5 nm with narrow particle size distribution. A detailed description of the synthesis process is found in [41,42]. The setup includes a standard 2 kW 2.45 GHz microwave (Muegge Electronic, Reichelsheim, Germany) with a rotating TE11 cavity [43] and a 24 mm diameter reaction vessel. Ironpentacarbonyl, Fe(CO)_5_, is used as liquid and volatile precursor, a mixture of Ar/20 vol% O_2_ serves as reaction and plasma gas. The precursor is evaporated prior to the plasma zone and inserted with Ar-carrier gas into the plasma zone. Here, the molecules are dissociated and ionized, the chemical reaction takes place, and the particle formation occurs. The resulting powder is collected via thermophoresis on cooled walls and scraped-off after the process. The relevant reaction parameters are summarized in Table 1. The main difference is the microwave power, leading to different plasma temperatures. The two produced samples are named after the resulting plasma temperature, i.e., P200 and P520.

For comparison, two commercially available Fe_2_O_3_ nanopowders are integrated in the study: (i) Iron(III) oxide <50 nm particle size from Sigma-Aldrich, order number 544884-25g/Lot 05223EE, made by a gas-phase synthesis without detailed specification (Sigma-Aldrich, Taufkirchen, Germany); (ii) γ-Iron(III) oxide, 20–40 nm, 99% Maghemite, order number NO-0053-HP, from Iolitec (Heilbronn, Germany), without specification of synthesis process.

A Tecnai F20 ST (FEI, now Thermofisher Scientific, operated at 200 kV), equipped with a GATAN 1024 × 1024 Multiscan CCD camera and a GATAN parallel electron energy loss spectrometer (EELS), is used for the microstructural characterization. The sample preparation is done by simply dipping Cu-grids, coated with lacey carbon films (Plano, Wetzlar, Germany, order number S166-3; carbon films with large open areas), into the powder. This ensures to have softly agglomerated powder particles fixed on the edges of the holes so that investigations of the particles can be done without disturbing carbon film. Electron diffraction is acquired with a camera length of 970 mm. The EELS spectra were collected in the TEM-diffraction mode (a diffraction pattern is on the screen and an image is located at the spectrometer object plane), with an extraction voltage of 4.4 kV, gun lens 4, using a camera length of 200 mm, a spectrometer entrance aperture of 2 mm, and a dispersion of 0.3 eV. The sample thickness (expressed in MFP—mean free path) was in the same order of magnitude for both microwave plasma synthesized powders, so that EELS spectra are comparable.

X-Ray powder diffraction (XRPD) patterns were collected at the European Synchrotron Radiation Facility (ESRF) in Grenoble, France, to be processed for analysis in the reciprocal space, according to the Rietveld method, and in the real space, through the pair distribution function (PDF). Data for *Q*-space investigation (Rietveld and line profile analysis) were collected at the high-resolution powder diffraction beamline (ID22), using the crystal analyzer setup, at incident wavelength *λ* = 0.4000 Å at room temperature. Data for real space analysis were obtained by increasing progressively the acquisition time at high scattering angles, up to *Q*max ~23 Å^−1^. The size and strain analysis of the samples was carried out using the Williamson–Hall (WH) method [44]. Rietveld refinements were performed via the GSAS software (Los Alamos, NM, USA. Version November 2012) [45]. Further PDF data were gathered at the ID15 beamline at *λ* = 0.142 Å and using an image plate detector, which allowed to cover a wave-vector *Q* region up to *Q*max ~30 Å^−1^, at four different temperatures (*T* = 120, 180, 240, 295 K) for about 30 min of acquisition at each temperature. Empty capillaries were measured in the same conditions in order to be subtracted from each specimen experimental curve and to retrieve only the diffraction signal coming from the sample. 2D images integration was performed by Fit2D-12 software (version 12, ESRF, Grenoble, France) [46]. For both experiments, samples were filled into capillaries with 1.5 mm diameter and rotated during acquisition. PDF data were reduced using pdfgetX3-1.1 (version 1.1., Trustees of Co lumbia University, New York, NY, USA) [47] and real space refinements were carried out by PDFgui-1.0 (version 1.0. Board of Trustees of Columbia, New York, NY, USA) [48].

X-ray absorption spectroscopy (XAS) investigations were carried out at room temperature and in transmission geometry at the Fe K-edge at the SUL-X beamline at ANKA (Karlsruhe, Germany). The four γ-Fe_2_O_3_ specimens plus a hematite standard were recorded. The SUL-X beamline delivers a focused, monochromatic beam by using a wiggler source, focusing mirrors and a cryogenically cooled Si(111) double crystal monochromator. The energy was calibrated using Fe foil in series to the sample and fixing its first derivative peak at 7112.0 eV [49]. Two scans were collected for each sample for averaging and to check reproducibility. The EXAFS data were reduced using the Demeter-0.9 package standard procedures (version 0.9.26, Naval Research Laboratory, Washington, DC, 20375, USA) [50]; fits of the *k*^2^ weighted EXAFS data were carried out in *r* space using theoretical functions from the FEFF9 code(version 9.6.4. The Feff project, Department of Physics, University of Washington, Washington, DC, USA) [51]. High-quality *k*-space data were obtained up to 14 Å^−1^ and the full fit range was 1.00–3.91 Å. Preliminary results are reported in [52].

Electron magnetic resonance (EMR) spectra were collected at a Bruker ELEXSYS spectrometer (Bruker, Billerica, MA, USA) equipped with an ER4102ST standard rectangular cavity at X band (9.4 GHz) frequency in the 130 ≤ *T* ≤ 410 K temperature range. The derivative d*P*/d*H* of power *P* absorbed was recorded as a function of the static magnetic field *H*.

The magnetic properties were studied using a quantum design (Quantum Design Inc., San Diego, CA, USA) superconducting quantum interference device (SQUID) magnetometer operating in the 5–300 K temperature range (maximum magnetic field *H* =50 kOe). For this kind of analysis, we focused on the P200, P520 and Iolitec samples.

Classical molecular dynamics simulations of maghemite bulk and nanoparticles were performed using the LAMMPS code [53]. We used the Born–Mayer force interatomic potential with the same empirical parameters employed to model hematite nanoparticles in Reference [54]. The potential was fitted to the density functional theory (DFT) equation of state of bulk α-Fe_2_O_3_, γ-Fe_2_O_3_ and ε-Fe_2_O_3_. In order to test the accuracy of the potential, we calculated the caloric curve (internal energy vs temperature) of bulk maghemite. We started from a 3 × 3 × 1 supercell of ordered maghemite (*P*4_1_2_1_2 space group) and from a 3 × 3 × 3 supercell (*P*4_3_32 space group) with site disorder. We performed constant temperature and pressure runs using the Nosé–Hoover method and with a timestep of 0.5 fs, from 300 K to 1200 K. For each temperature we equilibrated the system for 50 ps and computed the thermodynamic averages for 100 ps. The internal energy and the average volume per unit formula of bulk ordered and disordered maghemite as a function of temperature are shown in the supporting information, Figures S1 and S2. In the whole range of temperature, the ordered structure is more stable than the disordered structure by ~0.012 eV/atom. The volume of the ordered structure is smaller than the one of the disordered structure by ~0.4%. At temperatures higher than 900 K, we observed Fe ions migration from a tetrahedral site to a neighboring vacant octahedral site, as shown in the supporting information, Figure S3.

## 3. Results and Discussion

Before showing the results, let us first introduce the two reference structures for this study, i.e., magnetite and maghemite. Magnetite Fe_3_O_4_ exhibits the AB_2_X_4_ spinel structure, space group *Fd*-3*m*, where X forms the cubic close packed structure; A and B ions occupy half of the tetrahedral interstitial and half of the octahedral sites, respectively. Two non-equivalent Fe sites are present: Fe1 at (1/8, 1/8, 1/8) in a tetrahedral environment (A) while Fe2 lies at (½, ½, ½) in an octahedral environment (B). Oxygen ions are in (*x*, *x*, *x*) with *x* ~ ¼. Tetrahedra connect only to octahedra, sharing corners, while octahedra are joined by edges. For an exhaustive discussion on the spinel structure, see [55].

The structure of maghemite differs from magnetite by the presence of octahedral iron vacancies. In fact, the formula of maghemite can be rewritten as Fe_8_V_FeO_O_12_, where V_FeO_ is a vacancy of octahedral iron. In respect to magnetite, 1/6 of octahedral iron sites of maghemite are empty. The consequent ordering of Fe vacancies decreases the symmetry, according to the following group-subgroup relations:

*Fd*-3*m* → (*F*4_1_32) → *P*4_3_32 → *P*4_1_2_1_2 (*c*’ = 3*a*)

When vacancies form and (partially) order the structure is primitive cubic, space group *P*4_3_32; in this structure octahedral iron ions occupy two non-equivalent sites of multiplicity 12 and 4, the latter is partially empty (occupational factor ~ 0.33). In the following, we will refer to this phase as “disordered maghemite”. XRPD patterns of magnetite and disordered maghemite are very close to each other but they differ in the cell parameter (*a* = 8.397 Å and *a* = 8.346 respectively) and for the presence of superstructure peaks in the latter, which are due to the different extinction rules of the involved space groups. Four different crystallographic sites exist for oxygen ions. Further iron vacancies ordering causes the tripling of the *c* axis (*c*’~3*a*,) and the transition to the tetragonal *P*4_1_2_1_2 space group [56,57,58,59,60].

We will refer to this phase as to “ordered maghemite”. In ordered maghemite there are three different fully occupied tetrahedral Fe sites, six different fully occupied octahedral Fe sites, and an additional empty octahedral site. Oxygen ions are distributed over twelve different crystallographic sites.

In both maghemite phases, the connectivity among Fe coordination polyhedra does not change in respect to magnetite. In respect to the disordered maghemite, in the pattern of the order phase additional superstructure peaks appear.

### 3.1. Electron Microscopy

**Figure 1:**
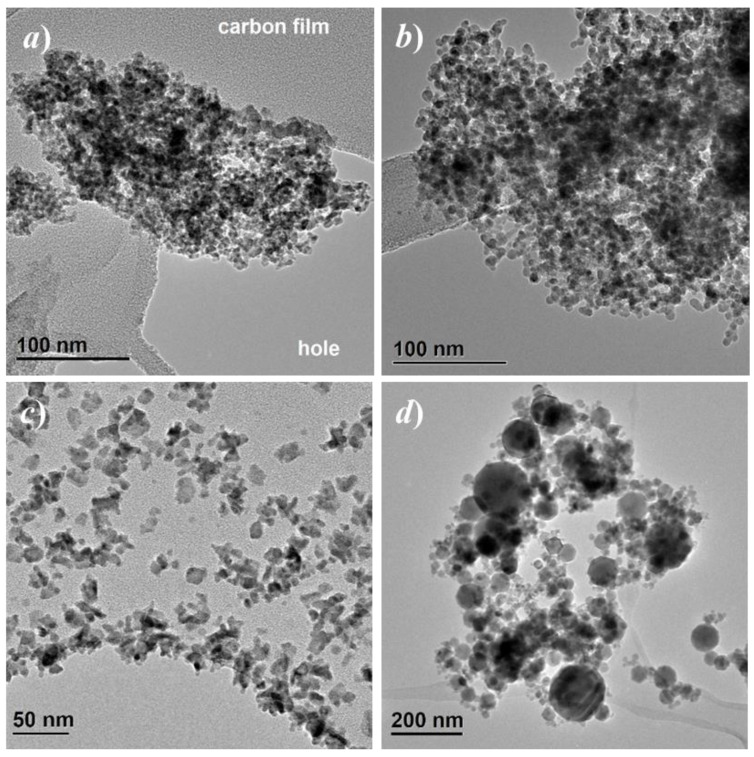
Bright field TEM images of specimens P200 (**a**) P520, (**b**) Iolitec, (**c**) and Aldrich (**d**).

TEM bright field images are reported in Figure 1. Further images with higher magnification are shown in the supporting information, Figure S4. Agglomerated particles with narrow particle size distribution centered well below 10 nm were observed for both P200 and P520, the P200 specimen showing smaller particles. The Iolitec specimen is characterized by mainly irregularly shaped particle, with a relatively narrow size distribution, around 15 nm. The Aldrich sample is instead characterized by a broader particle size distribution, with mostly spherical particles, ranging from a ten of nm to more than 100.

Electron diffraction images are reported in the supporting information, Figure S5. The diffraction signals suggest that both P200 and P520 are crystalline. In both cases, γ-Fe_2_O_3_ is the most probable phase. The diffraction rings indicate crystallite sizes in the nanometer range. The full width half maximum (FWHM) of the diffraction rings from specimen P520 appears slightly narrower than specimen P200. This indicates a larger crystallite size for P520, which is in agreement with the TEM bright field images. EELS spectra of specimens P200 and P520 are reported in the supporting information, Figure S6. In both cases the signal matches well those expected for γ-Fe_2_O_3_ maghemite.

### 3.2. X-ray Powder Diffraction

**Figure 2:**
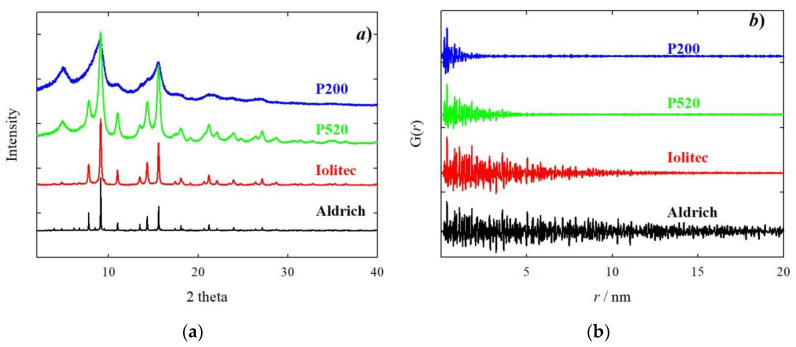
Experimental X-Ray powder diffraction (XRPD) patterns (**a**) and pair distribution function (PDF) curves (**b**) of Aldrich (black), Iolitec (red), P520 (green) and P200 (blue) samples. Experimental patterns have been renormalized to have similar scale.

The experimental high resolution XRPD patterns are shown in Figure 2a.

From the bottom to the top of the figure, a progressive broadening of Bragg reflections is observed. The origin of the peak broadening was investigated with the WH approach. A microcrystalline CeO_2_ reference sample was used to settle on the instrumental line profile broadening parameters. An example of WH plots is given in the supporting information, Figure S7. Table 2 sums up size and strain parameters. The peak broadening apparent in Figure 2a is related mainly to a finite size effect. According to the WH method, the samples P200 and P520 have crystal size as small as ~2 and ~4 nm, respectively. The trend of crystal size confirms TEM images (see Figure 1) though absolute values might be slightly different. This is due firstly by the fact that TEM provides a visual indication of size, and what looks like a single particle could be an aggregate. In the case of Aldrich sample, we have to consider that diffraction is a volume average technique, therefore the crystal size obtained (55 nm) is mediated over all the crystals and, most importantly, bigger crystals have a much larger weight. As to the microstrain, for the Aldrich sample it is limited compared to smaller nanoparticles. Reducing the size from Iolitec to P200, the microstrain increases, though to a lesser extent.

The ordered maghemite *P*4_1_2_1_2 phase is recognizable only in the Aldrich sample, where superstructure peaks typical of this phase are detectable (see SI, Figure S8). For the other samples, superstructure peaks were not detected. There are two possible reasons: (i) the broadening of the peaks hides the superstructure peaks or (ii) the ordered phase did not occur when the crystallite size becomes too small.

This is particularly true for specimens P520 and P200 which exhibit massive peak broadening. However, further tests with the tetragonal structural model did not improve the refinement. Having no evidence for the ordering of vacancies, for the other samples the maghemite phase is assumed to be disordered.

In addition, moving from P520 to P200, an increasing background contribution is observed in the synthesized samples. This could be consistent with the formation of an amorphous content, even though the very broad peaks can be modelled assuming the presence of very small and disordered hematite nanoparticles. We find the first option more plausible, as a similar amorphous effect was observed by Frison et al. [38] in the framework of magnetite-maghemite core-shell nanoparticles. Its origin was explained in terms of either a thin surface layer or the formation of subnanometric iron-oxo clusters.

Examples of Rietveld refinements are shown in the SI (Figure S9) and the corresponding structural parameters are listed in Table 3. However, we must underline that the massive peak broadening of specimen P200 might affect the reliability of the results.

In this respect, although maghemite and magnetite exhibit structure peaks with similar intensity and position, the lattice parameter can be taken as a reference to distinguishing them. Indeed, the oxidation from magnetite to maghemite induces a slight lattice contraction, typically from ~8.39 Å to ~8.34 Å, even though the absolute value can be affected by the small crystal size as well. As an example, Cervellino et al. [61] demonstrated a systematic expansion of magnetite unit cell for very small nanocrystals. Our specimens show lattice parameter of ~8.34 Å, therefore we can rule out the presence of magnetite, in keeping with EELS results. According to the cell parameters reported in Table 3, no trend is observed as a function of the crystallite size. Figure S10 in the supporting information compares the renormalized experimental patterns across the main maghemite peak. Only a slight shift seems to be observed for the P200 specimen, suggesting a minor volume expansion. However, this is not evident in the refined lattice parameters probably because the large peak broadening, and overlap, reduces the precision of the computed cell parameter.

Beside phase composition, the structural characterization is focused on the estimation of the iron sites occupancies, that we obtained by assuming the full stoichiometry to Fe_2_O_3_. Whereas the octahedral sites are in general roughly fully (Fe2, 12d) and half (Fe3, 4b) filled, reducing the crystal size induces the formation of vacancies on the tetrahedral (Fe1) 8c site. Let us first consider the passage from Iolitec to P520. The occupancy of the tetrahedral site decreases from ~95% to ~85% and is counterbalanced by the increased population of octahedral sites. Reducing the crystallite size to specimen P200 induces further modifications in the site occupations, but we believe that the computed structural parameters for very small nanocrystals might be significantly affected by the peak broadening, thus leading to underestimated uncertainties in Table 3. This prompted us to use local probes such as X-ray absorption spectroscopy and pair distribution function.

### 3.3. Pair Distribution Function

PDF data were collected using two different configurations. The high-resolution (HR) setup of the ID22 beamline of the ESRF allowed to obtain a significant PDF signal on a wide interatomic distance range, because the PDF intensity decay and broadening owing to limited instrument resolution are negligible [62]. Faster PDF data as a function of temperature were collected at the ID15 beamline of the ESRF using a 2D detector.

Figure 2b shows the experimental PDF using the HR setup. The decreasing size is evident in the damping of the PDF signal. Considering a negligible instrument effect, the crystal size can be estimated as the *r* value where the PDF signal goes to zero. Crystal size values can be extracted through a fitting procedure, considering a spherical envelope function [63]. The fits are shown in Figure S11. PDF sizes compare well with WH values (see Table 2), with the PDF providing slightly larger values for specimens P520 (5.4 against 3.8 nm) and Iolitec (13 nm against 11 nm). Examples and discussions of comparisons of crystal size extracted from PDF and different XRPD methods can be found elsewhere [64,65].

**Figure 3:**
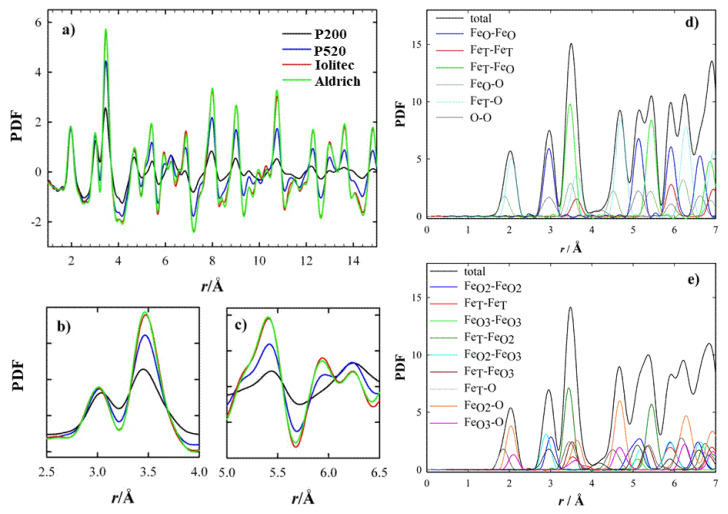
(**a**–**c**) Experimental PDF curves in different r ranges. (**d**,**e**) Calculated partial PDF according to magnetite (F*d*-3m) and maghemite (*P*4_3_32) structural models, respectively.

The temperature-resolved PDF collected with the 2D setup are displayed in the supporting information, Figure S12. The PDF peaks have scarce dependence on temperature: only small broadening is apparent by increasing temperature. Hence, we chose the lowest temperature (120 K) to compare the local scale of different specimens. This is reported in Figure 3a. The intensity of the peak at ~2.0 Å, which corresponds to the shortest Fe-O distances, is superposed for all the samples. As expected, by increasing the interatomic distances, the PDF peaks vanish more rapidly for samples with the smallest crystallite dimensions. However, not all the peaks are affected the same. In Figure 3b and 3c two *r*-regions are shown. Whereas the amplitude of the peaks at ~3.0 Å (Figure 3b) is nearly unaffected by the crystallite dimension, the one at ~3.5 Å decreases steeply for samples P520 and P200. This effect is accompanied by peak broadening. The same applies also to other specific atom pairs (5.1, 5.3 and 5.8 Å, see Figure 3c) at larger interatomic distances, while the opposite stands at 6.2 Å. In general, the smaller the crystallite size, the larger the peak width, suggesting an increased positional disorder.

**Figure 4:**
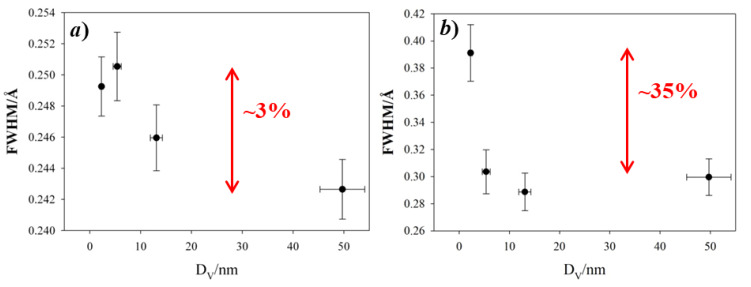
Full width half maximum (FWHM) of PDF peaks at ~3.0 Å (**a**) and ~3.5 Å (**b**) is plotted against the crystal size, with reference to the 120 K data collection. The gap of FWHM with respect to specimen Aldrich is highlighted in red.

The partial PDF approach can be used to visualize in a simple way which atom pairs contribute to each PDF peak. If we consider the experimental PDF (Figure 3a–c), the peaks most affected by the crystal size are those at ~3.5, ~5.3 and ~5.8 Å. The common feature to all these peaks is the involvement of Fe tetrahedral sites (hereafter Fe_T_), while the other pairs considered involve only octahedral Fe_O_ and O sites. This effect is quantified in Figure 4, where the peak width obtained by single peak fitting for the atom pairs at ~3.0 Å (left) and ~3.5 Å (right) is plotted against the crystal size, with reference to the data collection at 120 K. This suggests that reducing the crystal size produces a general increase in interatomic distance distribution, evidence of static disorder. This feature is however magnified for the atom pairs involving tetrahedral coordination: taking the Aldrich sample as a reference, for the smaller nanoparticles the broadening of the PDF peak at 3.5 Å increases by ~35%, while the peak at 3.0 Å increases only by ~3%.

As the same sites exhibit a lower peak intensity as well, a general reduction of the occurrence of tetrahedral site is attained. In order to prove this, we performed real space refinements in the 1.7 < *r* < 10 Å interval using a disordered maghemite structure model against low temperature (120 K) PDFs.

**Figure 5:**
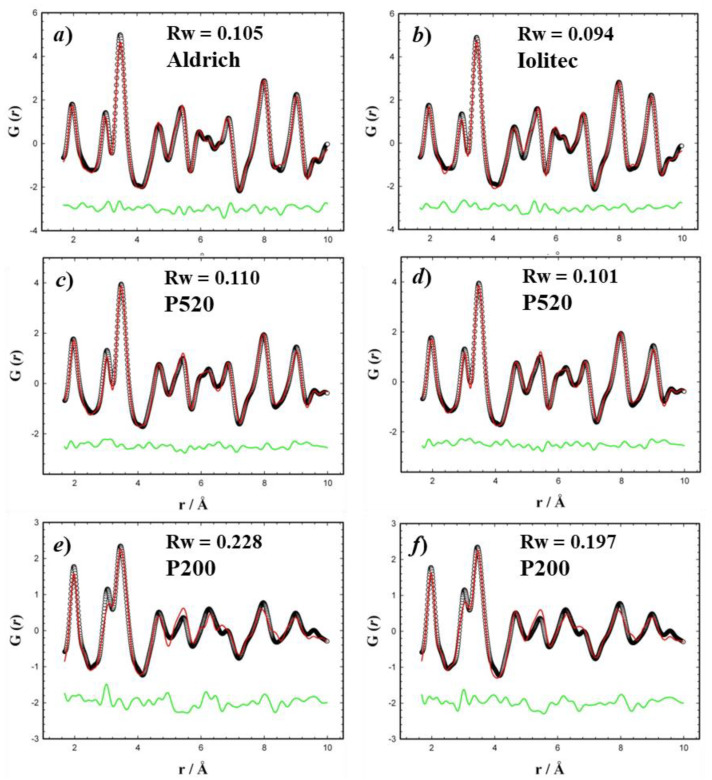
Real space Rietveld refinements of specimens (**a**) Aldrich, (**b**) Iolitec, (**c**,**d**) P520, (**e**,**f**) P200. The disorder maghemite structure was used in (**a**–**c**,**e**), the refinement of relative tetrahedral and partially occupied octahedral sites was allowed in (**d**,**f**).

The fits are shown in Figure 5. The structural model fits well the experimental PDF for Aldrich and Iolitec samples while a poorer accord is obtained for P520 and P200. A better fit was obtained by acting on the occupancies of tetrahedral and of partially occupied octahedral sites, which were varied fulfilling Fe_2_O_3_ stoichiometry. While for Aldrich and Iolitec samples the occupancies of tetragonal iron site remained unaffected, they decreased to 0.91 and 0.82 for P520 and P200 sample respectively, improving the fit quality. Figure 3d–f reports the fits for the two samples. It should be noted that (i) attempts to vary the occupancy of the fully occupied Fe_O_ site did not improve the fit; (ii) the residual parameter Rw for the P200 samples is still relatively high (0.197), thus suggesting complex disorder in this last sample featuring a large surface/bulk ratio.

To summarize, the main effect of a finite crystal size on the structure of γ-Fe_2_O_3_ is the progressive disordering of iron vacancies, which occurs mostly on the tetrahedral Fe sites. Whereas the Aldrich sample retains the tetragonal “ordered maghemite” structure, decreasing crystal size (Iolitec) the cubic disordered maghemite structure emerges, with one partially and randomly occupied octahedral iron site; in the P520 and P200 specimens, iron vacancies are shared by both octahedral and tetrahedral iron sites with the latter occupancy decreasing by reducing the crystal size.

This contradicts earlier investigations which, in respect to bulk maghemite, reported that Fe vacancies form exclusively, or preferentially, at the octahedral sites [39,66,67]. On the other hand, the selective formation of vacancies on the tetrahedral sites was observed during an in-situ hydrothermal synthesis of maghemite nanoparticles [40]. In particular, similar PDF signals as ours were observed during the first stage of the formation of the nanoparticles, when their size was very limited. The fact that similar structural defects are observed after different synthesis routes highlights the role played by surface effects.

### 3.4. X-ray Absorption Spectoscopy

#### 3.4.1. XANES

**Figure 6:**
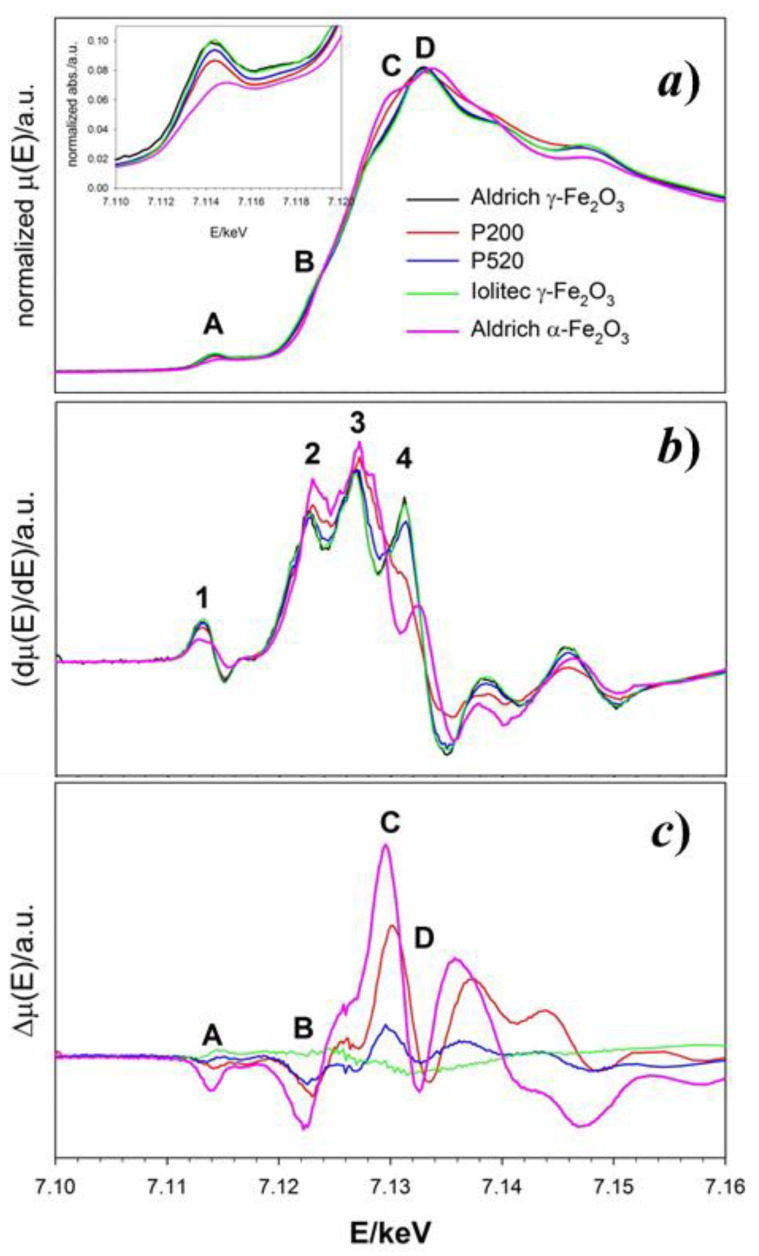
(**a**) Normalized absorption μ(*E*) spectra of maghemite and hematite samples around the Fe-K absorption edge. The Inset highlights the pre-edge peaks. (**b**) First derivative of the μ(*E*) curve for the same samples. (**c**) Difference Δμ(*E*) spectra obtained subtracting each μ(*E*) to the absorption spectrum of the Aldrich γ-Fe_2_O_3_ sample. In all panels, black, red, blue, green and pink curves refer to Aldrich, P200, P520, Iolitec γ-Fe_2_O_3_ and Aldrich α-Fe_2_O_3_ samples, respectively. Attribution of the A-D and 1–4 features are detailed in the main text.

The experimental absorption spectra μ(*E*) of all the maghemite γ-Fe_2_O_3_ and a reference hematite α-Fe_2_O_3_ samples across the Fe absorption K-edge are shown in Figure 6a. Four absorption peaks appear in the 7.11–7.35 keV range, labeled as A (~7.114 keV)**,** B (~7.122 keV), C (~7.130 keV) and D (~7.133 keV). Near edge features at higher energies will not be considered since they contain more multiple scattering contributions.

The first derivatives of the absorption with respect to the energy ∂μ∂E are displayed in Figure 6b. The derivative of the Aldrich γ-Fe_2_O_3_ sample (black line) shows four positive maxima (labeled 1 to 4) in correspondence to the inflection points of the μ(*E*) curve. Maxima 1 and 2 can be associated to the inflections points before peaks A and B of the μ(*E*), respectively. Maximum 3 corresponds to the edge energy (~7.127 keV); finally, maximum 4 drops in between peaks C and D. The positions and intensities of maxima 1–3 do not change significantly in the remaining maghemite samples. The intensity of maximum 4 decreases as the particle size decreases and it turns into a shoulder for P200 (red line). This is in agreement with the different shape of the absorption spectrum of P200. The derivative spectrum of the hematite standard matches that of maghemite only in correspondence to 2 and 3. Since the threshold position does not vary, the oxidation state of Fe is 3+ for all samples [68] and it appears to be unaffected by the crystallite size.

Figure 6c reports the difference spectra obtained subtracting the absorption spectrum of each sample to the reference Aldrich maghemite. Nanostructuring causes a progressive intensity decrease of peaks A and B on decreasing the particle size. The pre-edge A peak (see also the inset of the upper panel) corresponds to a forbidden quadrupolar Fe(1s→3d) transition which is permitted only in absence of inversion symmetry. The peak energy (7.114 keV) corresponds to transitions involving Fe^3+^. Indeed, a shoulder at lower energy (~7.111 keV) should appear in case of non-negligible Fe^2+^ ions concentration [69,70]. Its progressive intensity-decrease on reducing the particles dimension could be due to either a reduction of local structural distortion around Fe ions or to an increased concentration of Fe vacancies in tetrahedral sites.

Peak B at ~7.122 keV is present in all the spectra, including reference hematite, as confirmed by maximum 2 of the derivative spectra. Though being a common feature of Fe absorption K-edge spectra, to our knowledge only Berry and coworkers explicitly attributed it to a forbidden quadrupolar Fe(1s→4s) transition [70].

Peak D corresponds to the allowed dipolar electronic transition Fe(1s→4p), while C is attributed to the same transition coupled to a shakedown phenomenon due to a O(2p)→Fe(3d) transfer through a ligand-to-metal charge transfer process [71]. On nanostructuring, the difference spectra apparently show (i) a decrease of intensities of “forbidden” peaks A and B and (ii) an increase of intensity of peak C coupled to a decrease of D. The first trend matches to the smaller concentration of Fe_T_ in small nanoparticles, while the second could indicate an increased covalence character of the Fe-O bonding and/or an energy distribution of Fe(4p) states.

#### 3.4.2. EXAFS

Moving to the EXAFS region, data modeling using maghemite is unreliable due to the huge number of non-equivalent Fe ions. However, all the maghemite structures can be suitably derived from Fe_3_O_4_ magnetite by removing 1/6 of Fe ions in octahedral position, V_Fe_ ordering and consequent structural distortions. Thus, to perform the data fit, we started from a magnetite model modified on purpose, mainly in the coordination numbers, to include the first and second neighbors in the data. The percentage of tetrahedral (Fe_T_) and octahedral (Fe_O_) Fe sites was fixed to that of the ideal structure of maghemite (3/8 and 5/8, respectively).

We included in the fit the first Fe-O shell, and the four next nearest Fe-Fe, two for each Fe site geometry and the next Fe-O shell present only in the tetrahedral sites. Higher coordination shells and multiple scattering paths were not included. The initial fitting parameters were the Debye–Waller factors *σ*^2^, the interatomic bond lengths *R*, the *E*_0_ shift in the edge energy with respect to the theoretical value and the amplitude reduction factor *S*_0_^2^ from multielectron effects. The *E*_0_ shift was constrained to the same value for all the paths while *S*_0_^2^ was constrained for all the paths of each site geometry. The coordination numbers (CN) were fixed for all the shells employed in the fit. In order to reduce the correlations among parameters, the Debye–Waller factors and bond lengths separating Fe_O_ and Fe_T_ sites (3.473 Å) were constrained, leading to a single Fe_O_-Fe_T_ interatomic distance. An overview of all the fit results is given in Table 4 and in the supporting information, Figure S13a–d.

The nearest neighbor Fe-O distances as well as Fe_O_-Fe_O_ and Fe_T_-Fe_T_ increase on decreasing the crystal dimension as generally expected for oxide nanoparticles [61,64]. The Fe_O_-Fe_T_ distance varies within the standard deviation in Aldrich, Iolitec and P520 samples, while an unphysical short distance coupled to a huge σ^2^ is found for P200. This suggests that disorder exists between the two Fe substructures. Attempts to refine the relative amount of octahedral and tetrahedral Fe sites led to unphysically small values, though, in general, we observed a systematic decrease of Fe_T_ sites by reducing the particle dimension.

To improve the modelling of sample P200, which exhibits the smallest nanoparticles, we allowed lower coordination numbers of all the Fe-Fe and of the second tetrahedral Fe-O shells to account for external surface effects. New coordination numbers CN’ were set to CN’ = 2/3 CN apart for the first Fe-O shells. These alternative fit results are reported in Table 5 and in the supporting information (Figure S13e). Only the longest Fe-Fe distances are affected by the change in coordination numbers, while a general decrease of *σ*^2^ values is observed. Additionally, in this case, the largest *σ*^2^ value for the Fe_O_-Fe_T_ shell confirms the disorder.

### 3.5. Molecular Dynamics

We simulated the formation of maghemite nanoparticles with diameters of 2, 3, 4 and 5 nm, by classical molecular dynamics. We created the initial nanoparticles from a spherical cut of a bulk ordered maghemite and we increased its temperature from 300 to 1200 K in 10 ps. Then we slowly cooled the nanoparticles down to 100 K in 0.5 ns. Since maghemite is a metastable phase and cooling of a liquid Fe_2_O_3_ drop was shown to produce mostly ε-Fe_2_O_3_ nanoparticles [54], we kept the core of our nanoparticles at 100 K during all simulations. The diameter of the core was 1 nm for the 2-nm nanoparticle, and 1.6 nm for the larger ones. By this way, the solid core acts as a nucleation center for the crystallization of maghemite nanoparticles.

**Figure 7:**
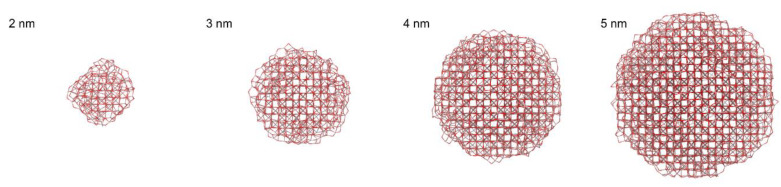
Maghemite nanoparticles generated by molecular dynamics, starting from a spherical cut of bulk maghemite of increasing diameter. Iron ions are pale red in color; oxygen ions are dark red.

The resulting nanoparticles generated by molecular dynamics are show in Figure 7. The maghemite bulk structure (i.e., the tetrahedral and octahedral sites) can be easily recognized in the large nanoparticles, while in the smaller nanoparticle (2 nm) the maghemite core is largely distorted, due to the large surface to bulk ratio. The shape of the smaller nanoparticles (2 and 3 nm) is roughly spherical, while the larger nanoparticles (4 and 5 nm) display flat facets of the {001} family, and stepped vicinals of the {001} surfaces. Furthermore, the generated nanoparticles display a large number of oxygen ions protruding outside and bridging with two or more iron ions. These bridging oxygens form high-strained small-membered rings. It is conceivable that in presence of moisture, these small-membered rings can break and react with water, forming hydroxyl groups.

Finally, we calculated the distribution of coordination numbers of the nanoparticles (see Table 6). The bond cut-off was chosen at 2.45 Å, corresponding to the first minimum after the first peak of the pair distribution function. The 2-nm nanoparticle show mostly 4-coordinated iron atoms, and a sizable amount of 3- and 5-coordinated Fe, which are absent in the bulk. The origin of the under-coordinated sites is mostly due to the dry surface termination, as we did not simulate surfaces terminated by–OH groups.

Overall, the ratio between 4-fold and 6-fold coordinated Fe is quite large (~2.5) because of the large number of surface sites. Upon increasing the size of the nanoparticle, the number of 6-fold coordinated sites increases relatively to the 4-fold coordinated ones. This is due to the relative increase of the bulk over the surface. However, the ratio between 4-fold and 6-fold coordinated sites remains larger than the ratio between tetrahedral and octahedral sites of bulk maghemite. Indeed 4-fold and 6-fold coordinated sites in calculations of dry particles have different meanings in respect to tetrahedral and octahedral crystallographic sites. As an example, a Fe ion in octahedral coordination in the ideal structure features smaller coordination number (Fe(III, IV or V)) if it lies at the surface of a nanoparticle. However, it still contributes to PDF peaks corresponding to Fe_O_ sites.

In order to estimate the impact of surface effects, we estimated the fraction of Fe ions placed within 2.5 Å from the surface. This results in ~60% of surface sites for 2 nm nanoparticles, which drops to ~45% already for 3 nm and down to 30% for 5 nm. This means that moving from specimen P520 to P200, the fraction of surface Fe surface sites nearly doubles.

**Figure 8:**
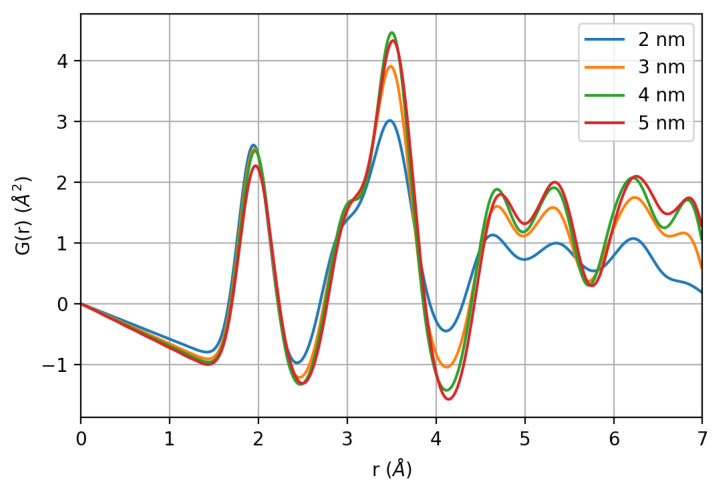
Pair distribution function of the nanoparticles generated by molecule dynamics.

The pair distribution function G(*r*) of the generated nanoparticles calculated using DiffPy-CMI-2.0 [72] is shown in Figure 8. Considering the very first coordination shells, up to ~4 Å, the trend recalls the experimental PDF. Indeed, whereas the first Fe-O neighbors and octahedral Fe-Fe pairs are nearly unaffected, the plot makes clear that surface relaxation effects can lead to a significant intensity decay of the peak at ~3.5 Å, as the one observed in experimental data (compare with Figure 3). The intensity decrease is about 1/3 compared to the 5-nm nanoparticles, while the next pair at 3.1 Å decreases only by ~15% and being a short distance, this variation cannot be considered as a simple size effect. Surface relaxation obviously contributes significantly to the observed peaks broadening.

### 3.6. Magnetic Properties

#### 3.6.1. EMR

**Figure 9:**
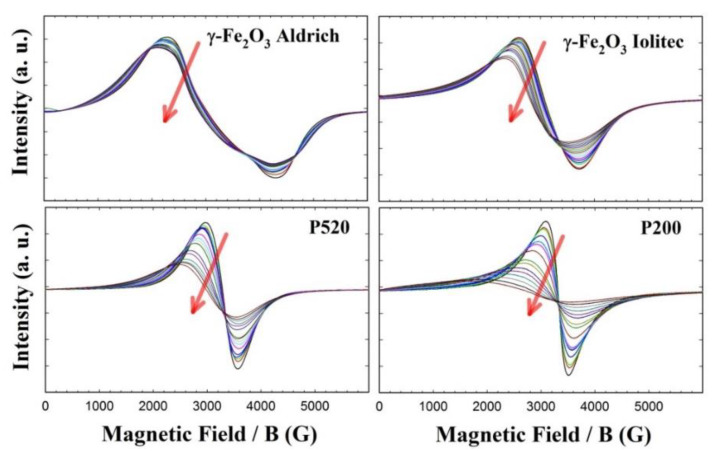
EMR spectra of γ-Fe_2_O_3_ samples in the 130–290 K range. Red arrows indicate cooling.

The EMR spectra of all the γ-Fe_2_O_3_ samples from 120 K up to room temperature are shown in Figure 9, while those collected at higher temperatures are given in the supporting information (Figure S14). The most apparent effect of decreasing the nanoparticle size is the flattening of the EMR signal at low temperature. Let us start the analysis from the Aldrich sample, which shows negligible surface effects compared to the other specimens. Its EMR spectra are composed of a broad band with a profile changing with temperature. The left part shifts toward lower magnetic field with decreasing temperature. This asymmetric line broadening, which is observed in all the samples, can be attributed to the raise of an internal field *H*a as it has been theoretically calculated in [73,74] and experimentally shown in [75,76,77]. It should be noted that in the case of a reduced relaxation time (*T*_2_) a symmetric broadening would be observed. The anisotropy field *H*a affects more markedly the resonance field profile at low temperature, while at higher temperatures it is almost completely cancelled by the thermal fluctuations [78].

Increasing temperature produces a symmetric narrowing of the spectral profile, and both left and right part of the spectrum shift toward the central field. However, the whole EMR pattern seems composed of many different contributions, so that a more detailed interpretation seems very difficult with this sample.

**Figure 10:**
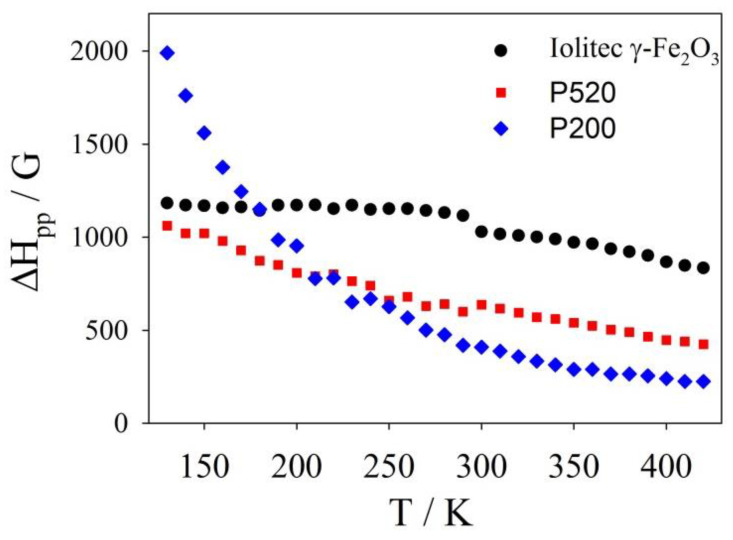
Δ*H*_pp_ values versus temperature as measured from EMR spectra for Iolitec, P520 and P200 samples.

Conversely, the EMR patterns of the Iolitec and P520 samples are composed of single lines, approximately Lorentzian-shaped and narrowing roughly linearly with increasing temperature in the whole temperature range (see Figure 9). The peak-to-peak line-width of the first-derivative spectrum (Δ*H*_pp_) is a powerful approach to extract significant information out of EMR spectra [79,80]. The temperature evolution of Δ*H*_pp_ is shown in Figure 10.

At low temperature, the line broadening is accompanied by a small shift toward lower fields of the left part of the profiles in the P520 sample. This can be attributed to the fact that, as the temperature decreases, the thermal energy contribution reduces and the effect of the internal field *H*a becomes more and more pronounced. Therefore, since the internal and external fields add up, the low-field lobe of the spectral pattern is observed at a lower applied field [73].

Similar cases were reported for superparamagnetic particles with diameters ranging between 16 and 23 nm, i.e., larger than those here examined [81]. A model was proposed assuming that at enough high temperature the anisotropy energy of the samples was much smaller than k_B_*T*, (k_B_ is the Boltzmann constant) namely of the thermal energy.

The P200 sample displays a Lorentzian shaped EMR spectrum and a linear trend of Δ*H*_pp_(*T*) curve only above ~ 300 K. Conversely, a highly asymmetric line broadening is observed on cooling at lower temperature (see Figure 9), accompanied by a large shift of the left part of the spectrum toward low field values and a steep increase of Δ*H*_pp_.

It follows that the internal magnetic field Ha at low temperature in specimen P200 is affected by a further contribution, which is otherwise negligible in the specimens with bigger crystals.

PDF and XAS results detected increasing structural disorder on reducing the crystal dimension, including changes in the occupational factors of octahedral and tetrahedral sites, which should be related to the increase of the surface-to-bulk ratio. We can assume that the very fine crystallites would consist of two parts: (a) an inner core whose magnetic moment can be aligned along the applied magnetic field; (b) a surface layer where magnetic moment cannot be turned entirely along the applied magnetic field, but makes an average canting angle with it. Indeed, a surface spin-canting was noticed with maghemite nanoparticles since at least the 1971 [82], with the canting thickness *t* increasing with decreasing *T* < *T*_B_ (the last being its “blocking temperature”), as well as with decreasing the maghemite particle size [83,84,85,86]. In this case, the thermal energy k_B_*T* becomes inadequate to overcome completely the anisotropy energy so that the EMR line is no more Lorentzian-shaped and its first derivative has a broader left lobe, as reported above.

#### 3.6.2. SQUID

**Figure 11:**
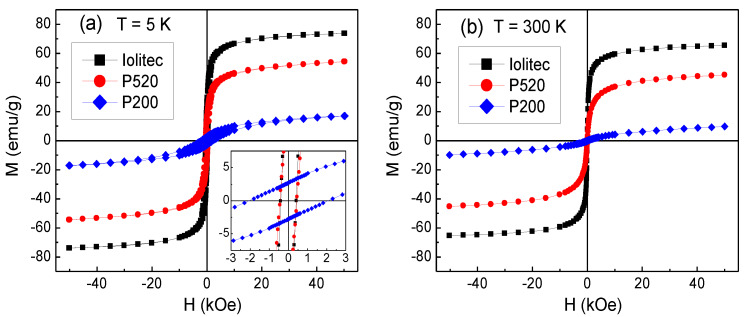
Magnetic hysteresis loops measured on the samples Iolitec, P520 and P200 at (**a**) *T* = 5 K (the inset is an enlarged view of the central region of the loops) and (**b**) *T* = 300 K.

Magnetic hysteresis loops of Iolitec, P520 and P200 samples measured at 5 K are shown in Figure 11a. The magnetization measured at *H* = 50 kOe decreases passing from the Iolitec sample to P520 and then to P200, namely with decreasing the nanoparticle size. Moreover, the loops exhibit a non-saturating tendency, which also becomes more pronounced for smaller crystals. The value of the saturation magnetization *M*_S_5K_ is extrapolated from the loops for 1/*H* tending to zero. The results are reported in Table 7.

*M*_S_5K_ is lower than the value of bulk maghemite *M*_S_bulk_ (~83 emu/g [87]) in all samples. The ratio *M*_S_5K_/*M*_S_bulk_ is about 92%, 71% and 27% for Iolitec, P520 and P200, respectively. A reduction of the saturation magnetization is generally observed in ferrite nanoparticles and mainly attributed to the spin canting, namely a lack of spin collinearity in the spinel structure, which also makes the approach to the magnetic saturation difficult.

The effect of spin canting is a hint of structural disorder, at the surface and/or in the core of the nanoparticles, since it generally arises because of modified atomic coordination and presence of topological defects, resulting in altered super-exchange bonds [17,23,82,88,89,90]. Therefore, the different values of *M*_S_5K_ indicate that the structural disorder affects the nanoparticles more and more on reducing their mean size. This is consistent with the effects of increase of Fe vacancies on the tetrahedral sites, bond distance expansion, loose of Fe-Fe connectivity between octahedral and tetrahedral sites—revealed by PDF and XAS analyses—which are more pronounced in smaller nanoparticles.

In this respect, it should be also considered the hypothetical presence of hematite in P520 and P200 as supposed by XRPD. Being hematite antiferromagnetic, its presence could contribute to depress *M*_S_5K_ and accentuate the non-saturating tendency in the hysteresis loops of those samples. The values of coercivity *H*_C_ at *T* = 5 K, obtained from the loops in Figure 11a, are comparable in Iolitec and P520, whereas *H*_C_ is substantially higher in P200 (Table 7).

Magnetic loops are also measured at *T* = 300 K and the results are shown in Figure 11b. The values of saturation magnetization *M*_S_300K_, extrapolated for 1/*H* tending to zero, are reported in Table 7. Compared to *M*_S_5K_, *M*_S_300K_ is reduced by about 11%, 17% and 40% in Iolitec, P520 and P200, respectively. Hence, a stronger thermal dependence of the saturation magnetization is experienced on decreasing the mean nanoparticle size, which is consistent with an increasing degree of structural and magnetic disorder. No magnetic hysteresis is observed at this temperature (i.e., the coercivity *H*_C_ and the remanent magnetization are null), which is in favour of a superparamagnetic behaviour of the magnetic nanoparticles.

**Figure 12:**
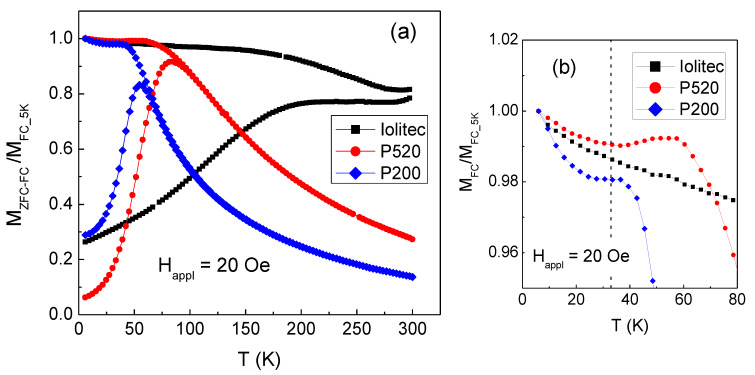
(**a**) Magnetization measured on Iolitec, P520 and P200 samples for increasing temperature, at *H*_appl_ = 20 Oe, after zero-field-cooling (*M*_ZFC_, lower branch of each displayed curve) and after field-cooling (*M*_FC_, upper branch). (**b**) The low-temperature part of the *M*_FC_ curves is displayed. The dashed vertical line marks the temperature *T* = 33 K (see text for explanation). In both frames, the curves are normalized to the value of M_FC_ at *T* = 5 K.

The magnetothermal behaviour of the samples is investigated by measuring the magnetization as a function of increasing temperature (heating rate 3 K/min) in a static magnetic field *H*_appl_ = 20 Oe, after cooling the sample from room temperature down to *T* = 5 K without *H*_appl_ (zero-field-cooling mode, ZFC) and in presence of *H*_appl_ (field-cooling, FC). Results are shown in Figure 12a, normalized to the value of M_FC_ at *T* = 5 K. The difference between *M*_FC_ and *M*_ZFC_ reveals the presence of thermally induced magnetic relaxation processes of the nanoparticle moments, which, in P520 and P200, culminate in a superparamagnetic behaviour at the irreversibility temperature *T*_irr_ at which the FC and ZFC branches join together; above *T*_irr_, the *M*_FC_ and *M*_ZFC_ curves are superposed and show a monotonic decreasing trend. Hence, *T*_irr_ is the highest blocking temperature of the nanoparticle assembly above which all the nanoparticles are in the superparamagnetic relaxation regime, in the adopted experimental conditions. In P520 *T*_irr_ ~ 88 K and *T*_irr_ ~ 70 K in P200. Moreover, it is worth noticing a particular feature of the FC curves of samples P520 and P200, not visible in Iolitec and whose origin will be explained later, i.e., the sudden increase of M_FC_ on decreasing temperature below ~ 33 K, highlighted in Figure 12b.

In the Iolitec sample, the ZFC and FC magnetization branches do not join up to *T* = 300 K. This indicates that, unlike P520 and P200 and in spite of the absence of magnetic hysteresis (Figure 11b), not all the nanoparticles have entered the full superparamagnetic state at this temperature.

The values of *T*_irr_ are much higher than expected for non-interacting maghemite nanoparticles as small as the ones we are considering. In fact, assuming the Néel expression for the relaxation time of the magnetic moment of a nanoparticle [91] and in the adopted experimental conditions (in SQUID magnetometry, the measuring time is assumed equal to 100 s), the blocking temperature *T*_B_ can be estimated using the relation *T*_B_ = *KV*/25k_B_, where *K*= 5 × 10^4^ erg/cm^3^ [87] is the magnetocrystalline anisotropy of bulk maghemite and *V* is the particle volume [92,93]. For a nanoparticle of the sample Iolitec, ~11 nm in size, *T*_B_ ~10 K is calculated and obviously lower values would be obtained for the P520 and P200 nanoparticles.

Actually, in the investigated samples the nanoparticles are not isolated, but they form large aggregates and even exhibit a tendency to coalesce, as observed in Figure 1. Therefore, the high values of *T*_irr_ are consistent with the existence of magnetic interactions among the nanoparticles, resulting in an increase of their anisotropy energy barriers for magnetization reversal and shifting to higher temperature or preventing their entrance in the superparamagnetic regime [91,93,94]. In other words, one can consider that the nanoparticles are subjected to an effective magnetic anisotropy *K*_eff_ higher than the anisotropy that operates when they are isolated.

For an assembly of non-interacting, single-domain and randomly oriented nanoparticles, the following relation holds: *H*_C_ = 0.96 *K*/*M*_S_, where *K* is the nanoparticle magnetic anisotropy and *M*_S_ is the saturation magnetization [95]. We use this relation to roughly estimate the effective anisotropy at *T* = 5 K, considering *K* = *K*_eff_ and setting *M*_S_ = *M*_S_5K_. Since in the above relation the magnetization must be expressed in (emu/cm^3^), we have multiplied the values of *M*_S_5K_ by the mass density of the samples, conventionally taken equal to that of bulk maghemite (5 g/cm^3^). The *K*_eff_ values obtained for the three investigated samples are reported in Table 7: they are definitely larger than the magnetocrystalline anisotropy of bulk maghemite; the values for Iolitec and P520 are comparable, whereas *K*_eff_ is substantially higher in P200.

**Figure 13:**
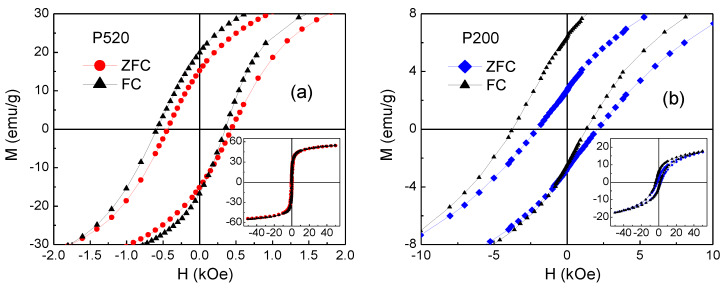
Hysteresis loops measured, at *T* = 5 K, in the ZFC mode (already shown in Figure 11a) and in the FC mode for sample (**a**) P520 and (**b**) P200. In the main frames, the central region of the loops is visualized; the whole curves are shown in the insets.

It is to be noted that if *K*_eff_ at T = 5 K were mainly affected by interparticle interactions of dipolar type, the highest *K*_eff_ should be found in the Iolitec sample, in line with the larger nanoparticle size and *M*_S_5K_ (Table 7). However, the opposite result is obtained. To elucidate this point, useful information has been obtained by measuring hysteresis loops at *T* = 5 K after cooling the samples from room temperature in an applied field *H*_cool_ = 50 kOe (FC mode). In Figure 13, the FC loops for samples P520 and P200 are displayed together with those already shown in Figure 11a, measured after cooling the samples without *H*_cool_ (ZFC mode). The FC loops are horizontally shifted towards negative field values, an effect known as exchange bias (EB), which originates from the exchange interaction at the interface between two different magnetic phases, structured on the nanometric scale [96,97,98,99].

In particular, it has been reported in spinel ferrite nanoparticles that the effect may arise due to the interaction between the ferrimagnetic core and a structurally disordered surface region, showing spin-glass-like magnetic behaviour [17,100,101,102]. This occurs through the following mechanism. The field *H*_cool_, applied above the freezing temperature of the glassy phase, aligns the magnetic moments of the nanoparticle cores along its direction. By cooling the nanoparticles across the freezing temperature, a spin configuration of the glassy phase is selected through the exchange coupling with the core moments. In turn, due to this exchange coupling, a preferential direction for the core moments is established, corresponding to that of *H*_cool_, namely a unidirectional anisotropy for the core moments appears (exchange anisotropy). Hence, the exchange anisotropy causes the loop shift, which is measured by the exchange field parameter *H*_ex_ = −(*H*_right_ + *H*_left_)/2, *H*_right_ and *H*_left_ being the points where the loop intersects the field axis. The shift of the FC loop is usually accompanied by an increase in the coercivity—defined as *H*_C_FC_ = (*H*_right_-*H*_left_)/2—with respect to that measured in ZFC mode [103,104].

The values of *H*_ex_ and *H*_C_FC_ measured in P520 and P200 are reported in Table 7. Compared to P520, sample P200 exhibits a stronger EB effect and a higher coercivity enhancement. No EB effect is observed in the Iolitec sample. Martinez et al. reported about a surface spin-glass transition in maghemite nanoparticles with mean size ~9 nm [100]. In particular, in a ZFC-FC magnetization measurement, they observed a sudden increase in *M*_FC_ below *T* ~42 K—hence, similar to that visible in P200 and P520 (Figure 12)—which they indicated as the freezing temperature of the surface spins.

Since, in our samples, the small nanoparticles form aggregates and tend to coalesce and considering that SQUID magnetometry is a volume sensitive technique, it is difficult to distinguish between core and surface regions. Therefore, as far as this type of analysis is concerned, we prefer to model the sample as an inhomogeneous material consisting of small regions with a good degree of structural and magnetic order, bearing a net ferromagnetic moment, embedded in a highly structurally and magnetically disordered matrix.

Thus, we provide the following description for the magnetothermal behaviour of the P520 and P200 sample with reducing temperature from 300 to 5 K. At high temperature, both the ferromagnetic moments and the spins of the disordered phase thermally fluctuate and the whole system behaves as a (super)-paramagnet. With reducing temperature, thermal effects reduce and below *T*_irr_ the ferromagnetic moments block progressively under the action of an effective anisotropy, namely along directions determined by a complex mix of magnetic interactions. In fact, it is to be expected that the ferromagnetic moments interact dipolarly and, moreover, as they get blocked they may exert a polarizing action on the spins of the disordered phase, which may be able to transmit the exchange interaction to neighbouring ferromagnetic moments [105]. Finally, with further reducing temperature below *T* ~33 K, the spins in the disordered phase undergo a collective freezing in a configuration determined by the competition between their own local anisotropy and the exchange interaction with the ferromagnetic moments, which leads to the onset of the exchange anisotropy.

Hence, with decreasing the nanoparticle size, namely passing from P520 to P200, the relative volume fraction of the ferromagnetic regions decreases, whereas the fraction of the glassy phase increases, which results in higher values of *H*_ex_ and *H*_C_FC_, in general agreement with literature results [103,106,107].

It is to be noted that at *T* = 5 K the ferromagnetic moments are locally subjected to exchange anisotropy also after zero-field-cooling, which accounts for the high values of *H*_C_, and thus of K_eff_, measured in P520 and particularly in P200 at *T* = 5 K. However, as the moments are randomly oriented, the exchange anisotropy averages out on a macroscopic scale and no EB effect is produced [108]. In the Iolitec sample no EB effect is observed and, in fact, the presence of an extended disordered phase with glassy magnetic character and able to trigger the exchange anisotropy is to be excluded, as indicated by the value of M_S_5K_, which is close to that of bulk maghemite. Therefore, in this case, we think that the magnetothermal behaviour of the nanoparticle assembly, including the value of *K*_eff_ at *T* = 5 K, is mainly affected by magnetic interactions of dipolar type and possibly of exchange type, in the case of intimate contact between the nanoparticles, but not mediated by the presence of an interfacial glassy magnetic phase.

## 4. Conclusions

Here we reported a comprehensive investigation on the structural and magnetic modifications induced by decreasing the crystal size of maghemite γ-Fe_2_O_3_ specimens. Crystal sizes were determined by XRPD and agreed well with TEM images. Except for the sample Aldrich, which exhibit a wide size distribution up to more than 100 nm, the other three specimens investigated have a relatively narrow size distribution, in the order of 10–15 nm for Iolitec, ~4–5 nm for P520 and ~2 nm for P200.

The main variations in structural properties occur when crystals are in the range of 2–5 nm, which unfortunately corresponds to the experimental conditions which hinder an accurate conventional diffraction investigation. Whereas the specimen with the larger crystals (Aldrich) exhibit evidence of tetragonal ordered maghemite structure; decreasing crystal size to ~10–15 nm likely stabilizes the cubic disordered maghemite. Although the superstructure peaks, fingerprint of the tetragonal phase are hard to resolve because of line broadening, the important disorder induced by surface effects is likely to prevent the ordering of iron vacancies.

Lattice parameters confirmed that the structure is actually maghemite and not magnetite for all the nanoparticles. This is also consistent with EELS spectra, whose peak energies are again characteristic of γ-Fe_2_O_3,_ and XANES, which confirmed that all samples show Fe only in the +3 state.

Rietveld refinements suggest that a crystallite size to ~5 nm induces the formation of iron vacancies on the tetrahedral site. However, the analysis for the smallest particles is hampered by the extensive broadening effects. This prompted us to use local probes. Low temperature PDF highlights a systematic structural disorder occurring for the smaller nanocrystals. In addition, the signal of the atom pairs involving tetrahedral sites exhibit a drastic intensity decay, while octahedral sites remained nearly unaffected. This is a further signature of the formation of vacancies on tetrahedral Fe sites, and it was further confirmed, and quantified, by real space Rietveld refinements. This agrees well with the recent findings by Jensen et al. [40], who observed that the formation of maghemite nanoparticles during hydrothermal synthesis starts from edge-sharing FeO_6_ octahedra, leading to Fe vacancies mostly on tetrahedral sites. It is also surprising to note that similar defects are obtained with different synthesis methods, thus suggesting that surface relaxations play a key role in the nature of the defects.

In addition, EXAFS on the Fe K-edge revealed that the atom pairs most sensitive to disorder are those connecting Fe ions in the two different coordinations. This effect becomes extreme in the case of ~2 nm nanocrystals.

Molecular dynamics simulations were carried out to estimate the evolution of Fe coordinations in very small maghemite nanoparticles. Important changes are predicted when reducing the size from 5 to 2 nm, increasing significantly the amount of undercoordinated Fe ions. This suggests a significant truncation of the coordination of the surface octahedral sites, leading to 4-fold, but also 5-fold, coordinated sites. This leads to important surface relaxations, which clearly impacts the smallest nanoparticles. The close similarity of the experimental and simulated PDF curves, especially in respect of the intensity of the peak at 3.5 Å, suggests that the increase of Fe vacancies on tetrahedral sites is intimately connected to surface disordering. Indeed, the simulated PDF reproduces well the features of the first coordination shells of experimental PDF and their trends on reducing particles dimension.

The study of the magnetic properties provides further evidence of a high degree of structural disorder for small nanoparticles. The EMR patterns of the Iolitec, P520 and P200 samples are composed of single lines, approximately Lorentzian-shaped which asymmetrically broaden shifting of the left part of the profiles toward lower fields on decreasing *T*. This effect is boosted for the P200 sample suggesting that surface disorder affects the alignment of magnetic moments along the applied field. The decrease of the saturation magnetization *M*_S_5K_, as revealed by SQUID measurements and its stronger thermal dependence with reducing the nanoparticle size are clear hints of a spin canting effect, determined by local deviations of atom positions from the average crystallographic structure. The structural disorder results in the appearance of a magnetic disorder phenomenology in the P520 and P200 samples. In fact, in these samples, the observation of exchange bias effect at *T* = 5 K has been explained considering the existence of essentially ferromagnetic regions exchange-coupled to a spin glass-like magnetic phase. The presence of this glassy magnetic phase and of interparticle dipolar interactions rule the magnetothermal behavior of P520 and P200. The latter features three different magnetic regimes: (i) superparamagnetic relaxation at high temperature, (ii) progressive blocking of the ferromagnetic moments with decreasing temperature below *T*_irr_ and (iii) collective freezing of the spins of the glassy magnetic phase below ~33 K, which, besides the exchange bias effect, also leads to high values of *H*_C_ and *K*_eff_.

## Figures and Tables

**Table 1:** Synthesis parameters.

Sample	Precursor Feed Rate	Microwave Power	Plasma Temperature	System Pressure
P200	15 mL/h	150 W	200 °C	8 mbar
P520	15 mL/h	1000 W	520 °C	8 mbar

**Table 2:** Crystallite size and microstrain computed by Williamson–Hall (WH) analysis, compared to crystallite and nanoparticle size estimated by PDF and TEM.

Sample	Strain WH	Dimension WH/nm	Dimension PDF/nm	Dimension TEM/nm
Aldrich	0.0022 (3)	55 (5)	50 (4)	~10–~100
Iolitec	0.0084 (4)	11.3 (1.0)	13 (1)	~15
P520	0.0084 (6)	3.8 (1)	5.4 (8)	<10
P200	0.0095 (7)	2.3 (1)	2.4 (3)	<5

**Table 3:** Crystallographic parameters computed by Rietveld refinements.

	Aldrich	Iolitec	P520	P200
Space Group	*P*4_1_2_1_2	*P*4_3_32	*P*4_3_32	*P*4_3_32
*a*/Å	8.3463 (1)	8.3531 (3)	8.3439 (11)	8.3464 (44)
*c*/Å	25.0269 (1)	-	-	-
*msd* (Fe)/Å^2^	0.0011 (1)	0.00778 (9)	0.00476 (4)	0.00141 (5)
*msd* (O)/Å^2^	0.0083 (4)	0.0150 (2)	0.00476 (4)	0.00141 (5)
Fraction Fe1(8c) (tetrahedral)	-	0.9458 (1)	0.841 (4)	0.8766 (5)
Fraction Fe2(12d) (octahedral)	-	0.9523 (1)	1	1
Fraction Fe3(4b) (octahedral)	-	0.586 (3)	0.653 (7)	0.5818 (8)
R (F^2^)	0.0448	0.0369	0.0734	0.0070

**Table 4:** Coordination numbers CN (fixed), Debye–Waller factors (s.d. ± 2%) and interatomic bond lengths *R* (s.d. ± 0.01 Å) determined from the EXAFS data analysis of the four maghemite samples. FeT and FeO refer to iron in tetrahedral and octahedral coordination, respectively. O’ refers to the second oxygen neighbor. The first atom of the pair stands for the absorber, the second for the backscatterer.

Pair	CN	P200		P520		Iolitec		Aldrich	
		*σ*^2^(Å^2^)	*R*(Å)	*σ*^2^(Å^2^)	*R*(Å)	*σ*^2^(Å^2^)	*R*(Å)	*σ*^2^(Å^2^)	*R*(Å)
Fe_T_-O	4	0.00154	1.869	0.00295	1.858	0.00170	1.826	0.00211	1.811
Fe_T_-Fe_O_	10	0.04892	3.161	0.02645	3.460	0.01535	3.461	0.01411	3.469
Fe_T_-O’	12	0.00424	3.395	0.00096	3.397	0.00070	3.369	0.00089	3.343
Fe_T_-Fe_T_	4	0.01133	3.888	0.00765	3.870	0.00510	3.841	0.00238	3.807
Fe_O_-O	6	0.00558	1.999	0.00615	1.971	0.00428	1.956	0.00163	1.957
Fe_O_-Fe_O_	5	0.01531	3.003	0.01062	2.982	0.00924	2.959	0.00894	2.956
Fe_O_-Fe_T_	6	0.04892	3.161	0.02645	3.460	0.01535	3.461	0.01411	3.469

**Table 5:** Coordination numbers CN’ (fixed), Debye–Waller factors (s.d. ± 2%) and interatomic bond lengths *R* (s.d. ± 0.01 Å) determined from the EXAFS data analysis of the P200 sample with the new CN’ values.

Pair	CN’	P200	
		*σ*^2^(Å^2^)	*R*(Å)
Fe_T_-O	4	0.00154	1.871
Fe_T_- Fe_O_	7	0.01711	3.867
Fe_T_-O’	8	0.00133	3.420
Fe_T_- Fe_T_	3	0.00574	4.051
Fe_O_-O	6	0.00567	1.999
Fe_O_-Fe_O_	3	0.00860	2.997
Fe_O_-Fe_T_	4	0.01711	3.867

**Table 6:** Fe coordination and amount of Fe ions within 2.5 Å from the surface as a function of maghemite crystal size expressed as (top) total and (bottom) relative (%) amount.

Diameter (nm)	Formula	Fe (III)	Fe (IV)	Fe (V)	Fe (VI)	Surface Fe Ions
2	Fe_155_O_236_	3	89	28	35	93
3	Fe_525_O_790_	10	256	83	176	238
4	Fe_1233_O_1844_	36	549	145	503	426
5	Fe_2404_O_3628_	39	993	292	1079	711
2	Fe_1.97_O_3_	1.9	57.4	18.1	22.6	60.0
3	Fe_1.99_O_3_	1.9	48.8	15.8	33.5	45.3
4	Fe_2.01_O_3_	2.9	44.5	11.8	40.8	34.5
5	Fe_1.99_O_3_	1.6	41.3	12.1	44.9	29.6

**Table 7:** Saturation magnetization *M*_S_5K_ and coercivity *H*_C_, measured at *T* = 5 K (in ZFC mode); saturation magnetization *M*_S_300K_, measured at *T* = 300 K; effective magnetic anisotropy *K*_eff_ calculated for *T* = 5 K; exchange field *H*_ex_ and coercivity *H*_C_FC_, obtained from the analysis of the FC loops at *T* = 5 K.

Sample	*M*_S_5K_ (emu/g) ± 2%	*H*_C_ (Oe) ± 5 Oe	*M*_S_300K_ (emu/g) ± 2%	*K*_eff_ (10^5^ erg/cm^3^) ± 3%	*H*_ex_ (Oe) ± 5 Oe	*H*_C_FC_ (Oe) ± 5 Oe
Iolitec	76.3	402	67.6	1.6	-	402
P520	58.6	440	48.7	1.3	118	468
P200	22.2	2062	13.2	2.4	1211	2542

## Supplementary Materials

The following are available online at https://www.mdpi.com/2079-4991/10/5/867/s1, Figure S1: calculated internal energy vs T, Figure S2: calculated cell volume vs T, Figure S3: MD snapshots, Figure S4: TEM images, Figure S5: electron diffraction, Figure S6: EELS spectra, Figure S7: WH plot, Figure S8: superstructure XRPD peaks, Figure S9: Rietveld refinements, Figure S10: XRRD maghemite peak, Figure S11: long range PDF refinements, Figure S12: temperature resolved PDFs, Figure S13: EXAFS refinements, Figure S14: high temperature EMR spectra.
