# Supplementary material for: Local Structure and Magnetism of Fe2O3 Maghemite Nanocrystals: The Role of Crystal Dimension
Source: Nanomaterials (Basel). 2020 Apr 30;10(5):867. doi: 10.3390/nano10050867 (PMC7279456; doi:10.3390/nano10050867)
Supplement: Supplementary file 1 [file nanomaterials-10-00867-s001.pdf]

Supporting information

Local Structure and Magnetism of Fe_2O_3 Maghemite Nanocrystals: The Role of Crystal Dimension

Mauro Coduri [1,*], Paolo Masala [2], Lucia Del Bianco [3], Federico Spizzo [3], Davide Ceresoli [4], Carlo Castellano [2], Serena Cappelli [2], Cesare Oliva [2], Stefano Checchia [5], Mattia Allieta [2], Dorothee-Vinga Szabo [6], Sabine Schlabach [6], Michael Hagelstein [7], Claudio Ferrero [8,†] and Marco Scavini [2,*]

1 Department of Chemistry, University of Pavia, viale Taramelli 16, 27100 Pavia, Italy
2 Department of Chemistry, University of Milan, via Golgi 19, 20131 Milano, Italy; masala1983@libero.it (P.M.); carlo.castellano@unimi.it (C.C.); serena.cappelli@unimi.it (S.C.); cesare.oliva@unimi.it (C.O.); mattia.allieta@gmail.com (M.A.)
3 Department of Physics and Earth Sciences, University of Ferrara; Via Saragat 1, 44122 Ferrara, Italy; lucia.delbianco@unife.it (L.D.B.); federico.spizzo@unife.it (F.S.)
4 National Research Council of Italy, Institute of Chemical Science and Technology (CNR-SCITEC), 20133 Milano, Italy; davide.ceresoli@cnr.it
5 Lund University, MAX IV Laboratory, 22100 Lund, Sweden; stefano.checchia@maxiv.lu.se
6 Karlsruhe Institute of Technology, Institute for Applied Materials (IAM) and Karlsruhe Nano Micro Facility (KNMF), Hermann-von-Helmholtz-Platz 1, 76344 Eggenstein-Leopoldshafen, Germany; dorothee.szabo@kit.edu (D.-V.S.); sabine.schlabach@kit.edu (S.S.)
7 Karlsruhe Institute of Technology, Institute for Beam Physics and Technology (IBPT), Hermann-von-Helmholtz-Platz 1, 76344 Eggenstein-Leopoldshafen, Germany; michael.hagelstein@kit.edu (M.H.)
8 European Synchrotron Radiation Facility, 38000 Grenoble, France; ferrero@esrf.fr
* Correspondence: mauro.coduri@unipv.it (M.C.); marco.scavini@unimi.it (M.S.); Tel.: +39-0382-987-212 (M.C.); +39-02-5031-4270 (M.S.)
† In memory of Dr. Claudio Ferrero.

Received: 30 March 2020; Accepted: 23 April 2020; Published: 30 April 2020

Molecular dynamics:

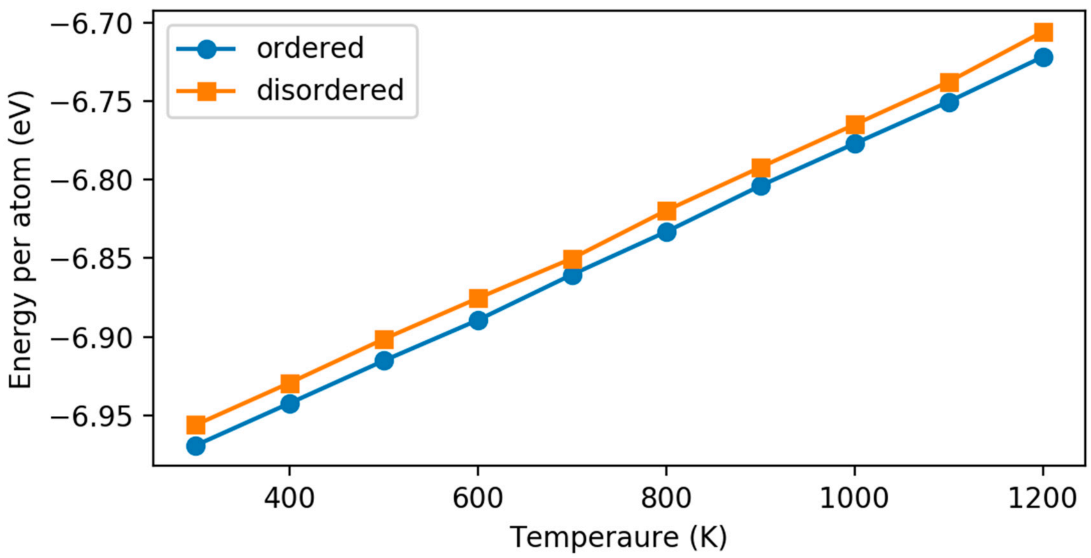

Figure S1. Calculated average internal energy as function of temperature for bulk ordered and disordered maghemite.

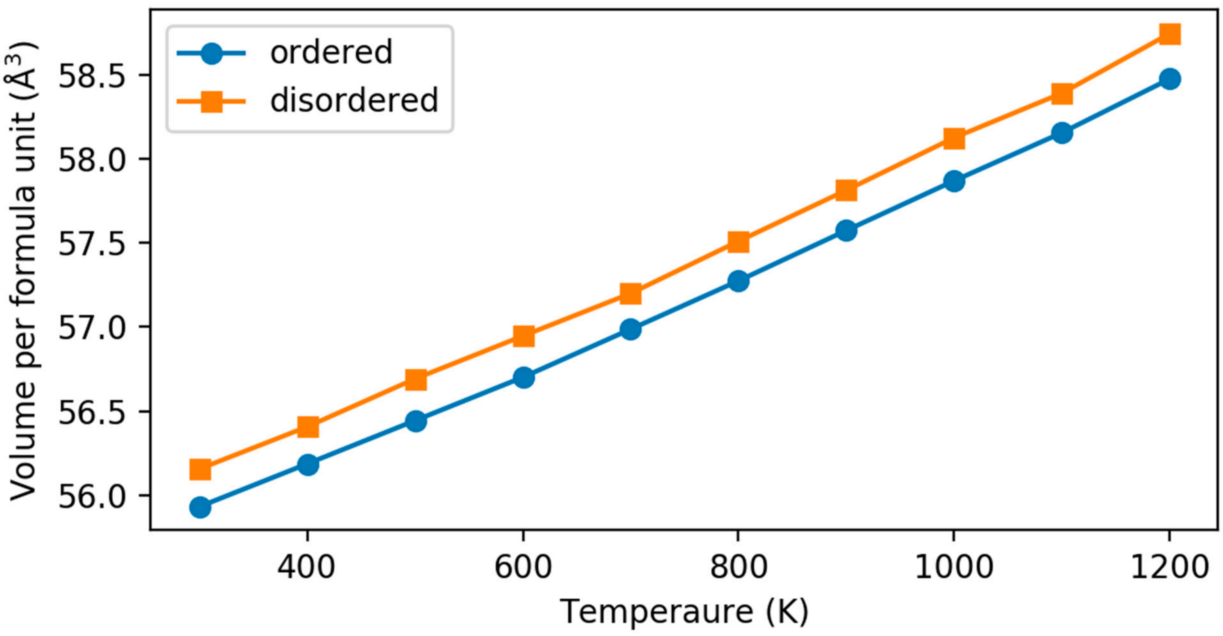

Figure S2. Calculated average volume per Fe_2O_3 as function of temperature for bulk ordered and disordered maghemite.

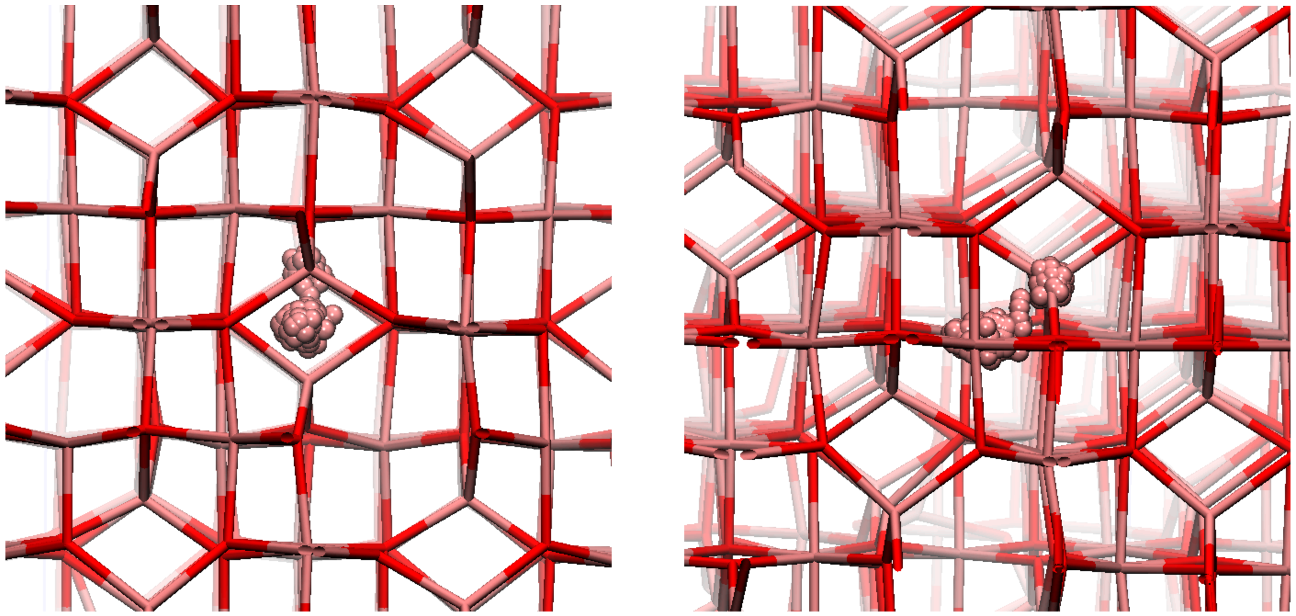

Figure S3. Molecular dynamics snapshots of the migration of an iron atom from a tetrahedral site to an octahedral site, at 1000 K. Left: view along the [110]. Right: view along the [010] direction. Iron ions are pale red in color; oxygen ions are dark red.

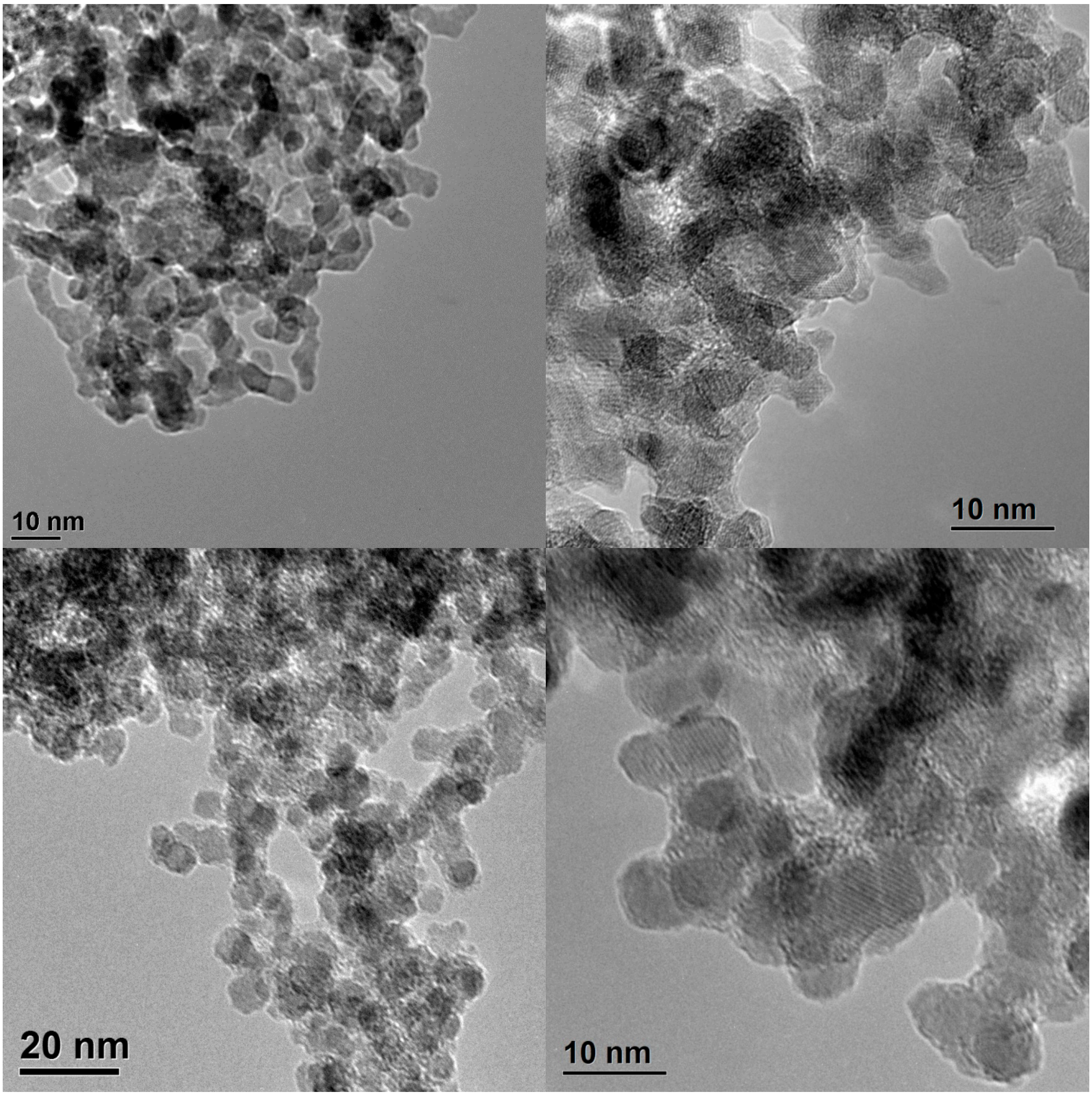

Figure S4. TEM bright field images with high magnification for specimens P200 (top) and P520 (bottom).

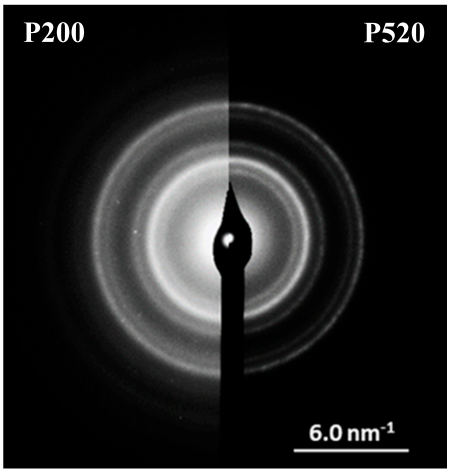

Figure S5. Electron diffraction for both microwave plasma synthesized samples.

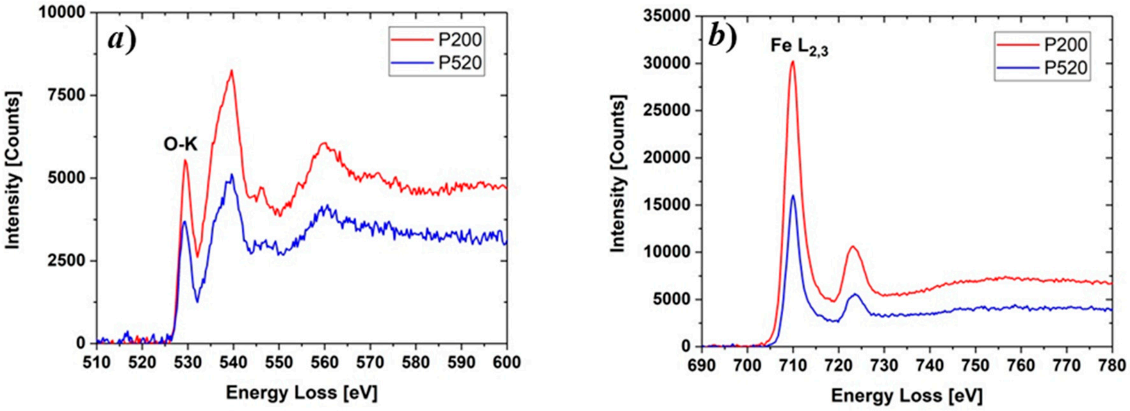

Figure S6. Background-subtracted EELS spectra at O-K (a) and Fe-$L_{2,3}$ (b) edges. The spectra for both microwave plasma synthesized materials are very similar. The O-K edge is composed of a prepeak slightly below 530 eV, a broader edge with maximum intensity at ~540 eV, a weaker maximum ~545 eV, and a major and broader contribution at ~560 eV. The energy difference between prepeak and main peak of the O-K edge is 9.6 ±0.3 eV for both materials. This is slightly lower than the values indicated in [S1] for γ-Fe_2O_3. The core losses at the Fe $L_{2,3}$ edge show also the typical and expected features for γ-Fe_2O_3. The energy difference of both maxima is 13.2 ±0.3 eV. This is in good agreement with Colliex et al. [S1].

[S1] Colliex, C.; Manoubi, T.; Ortiz, C. Electron-energy-loss-spectroscopy near-edge fine structures in the iron-oxygen system. *Phys. Rev. B* **1991**, *44*, 11402-11411.

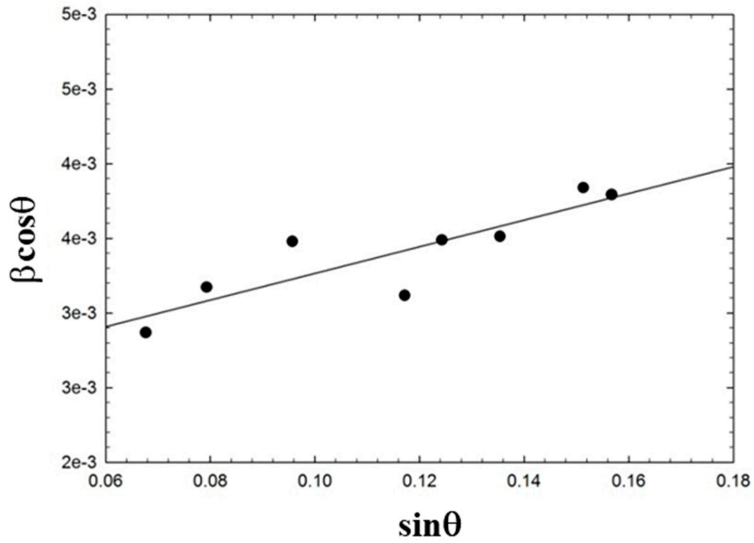

Figure S7. Example of Williamson Hall plot for sample P520.

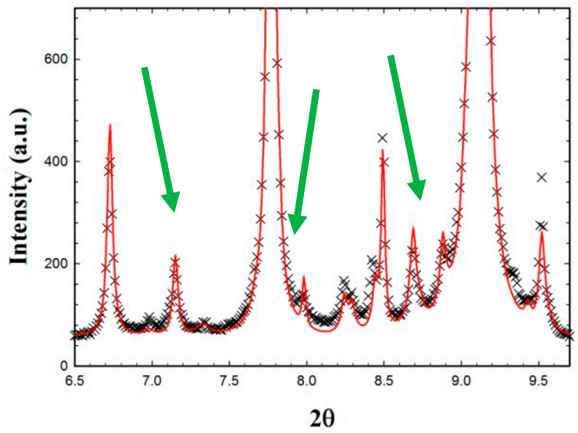

Figure S8. 2theta range from 6 to 10 of the Aldrich sample. The superstructure peak are highlighted by the arrows.

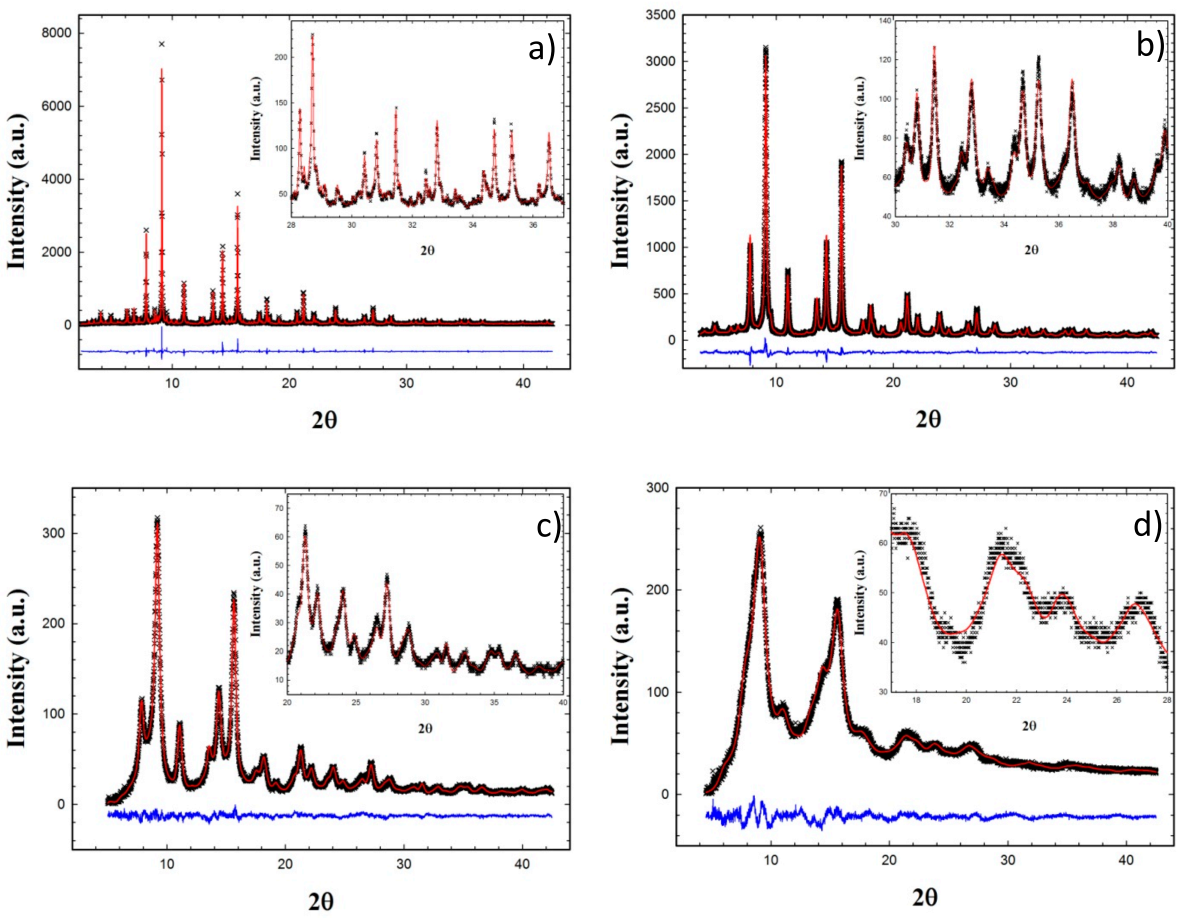

Figure S9. Rietveld refinements for Aldrich (a), Iolitec (b), P520 (c) and P200 (d) samples. Experimental pattern (black crosses), fit (red curves) and differential (blue line) are reported. In the insets the high 2theta range is highlighted.

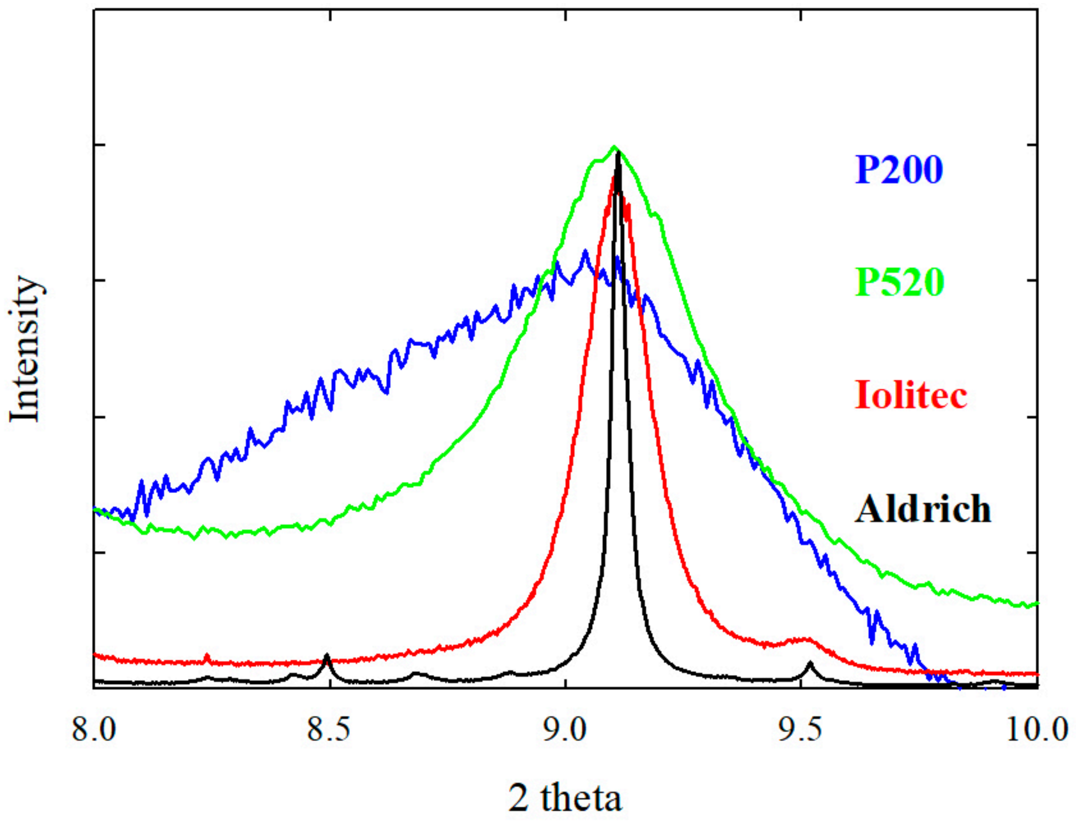

Figure S10: Normalized experimental patterns across the main maghemite diffraction peaks.

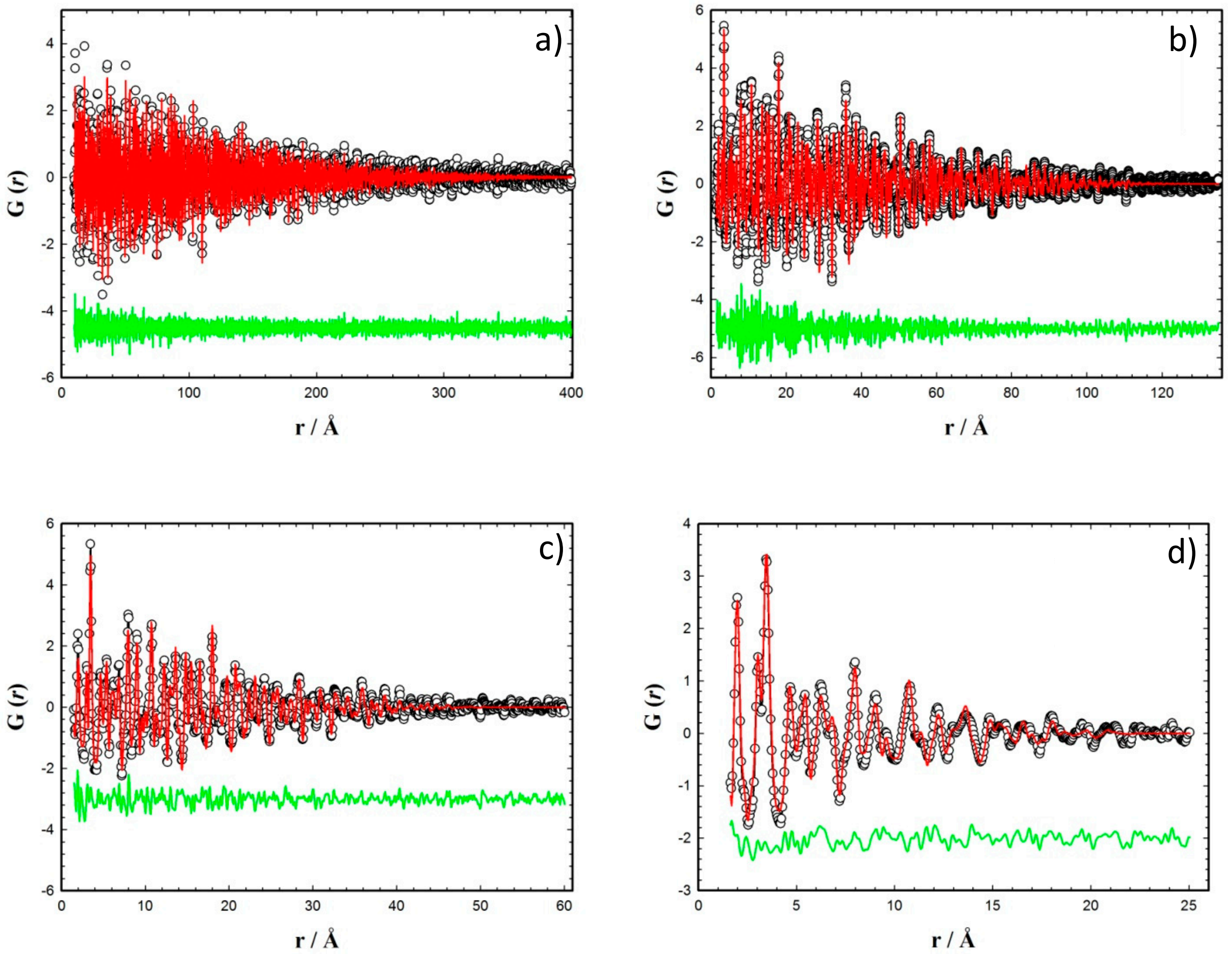

Figure S11. PDF refinements for Aldrich (a), Iolitec (b), P520 (d) and P200 (e) samples. Experimental pattern (black symbols), fit (red curves) and differential (green line) are reported.

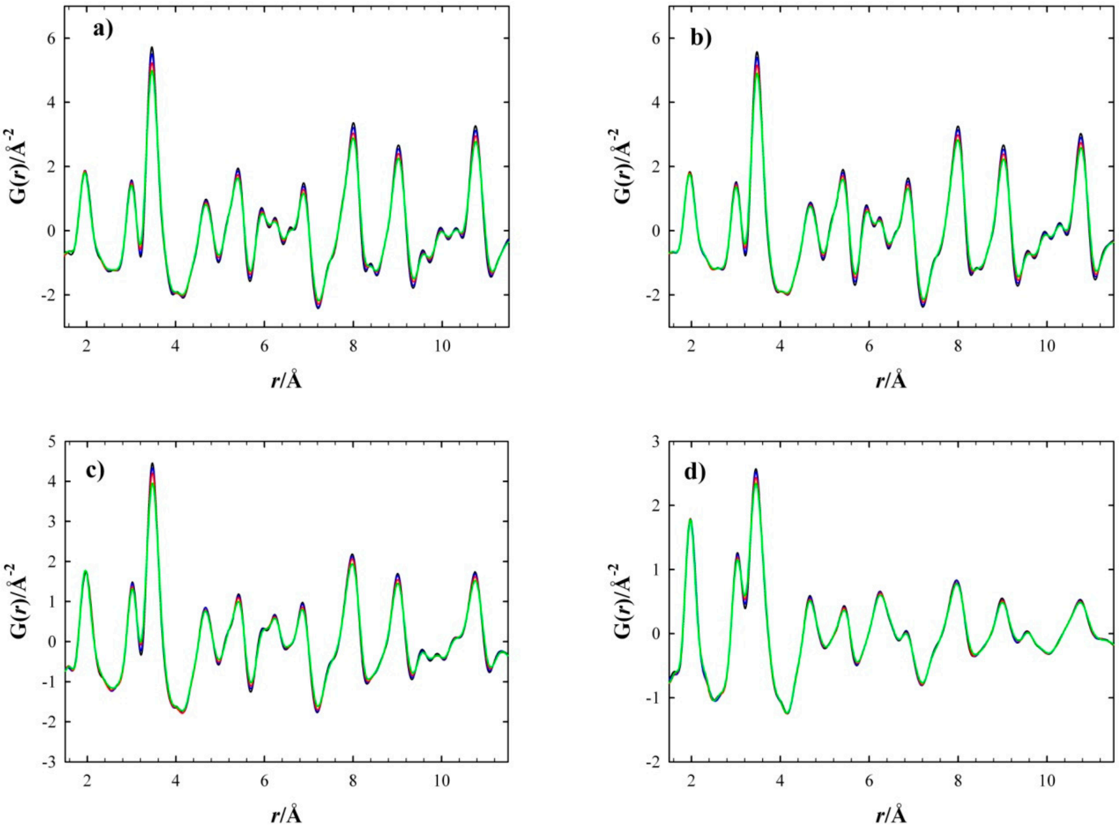

Figure S12. Experimental PDF of Aldrich (*a*), Iolitec (*b*), P520 (*c*) and P200 (*d*) samples at 120K (black), 180 K (blue), 240 K (red) and 295 K (green).

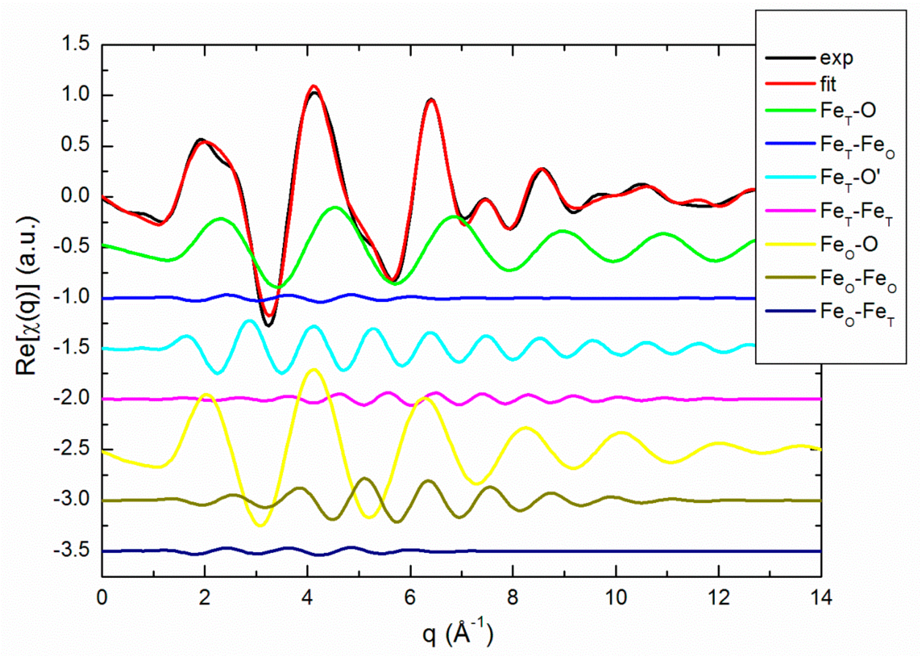

Figure S13a. Back Fourier transform best-fit curve and single contributions for sample P200.

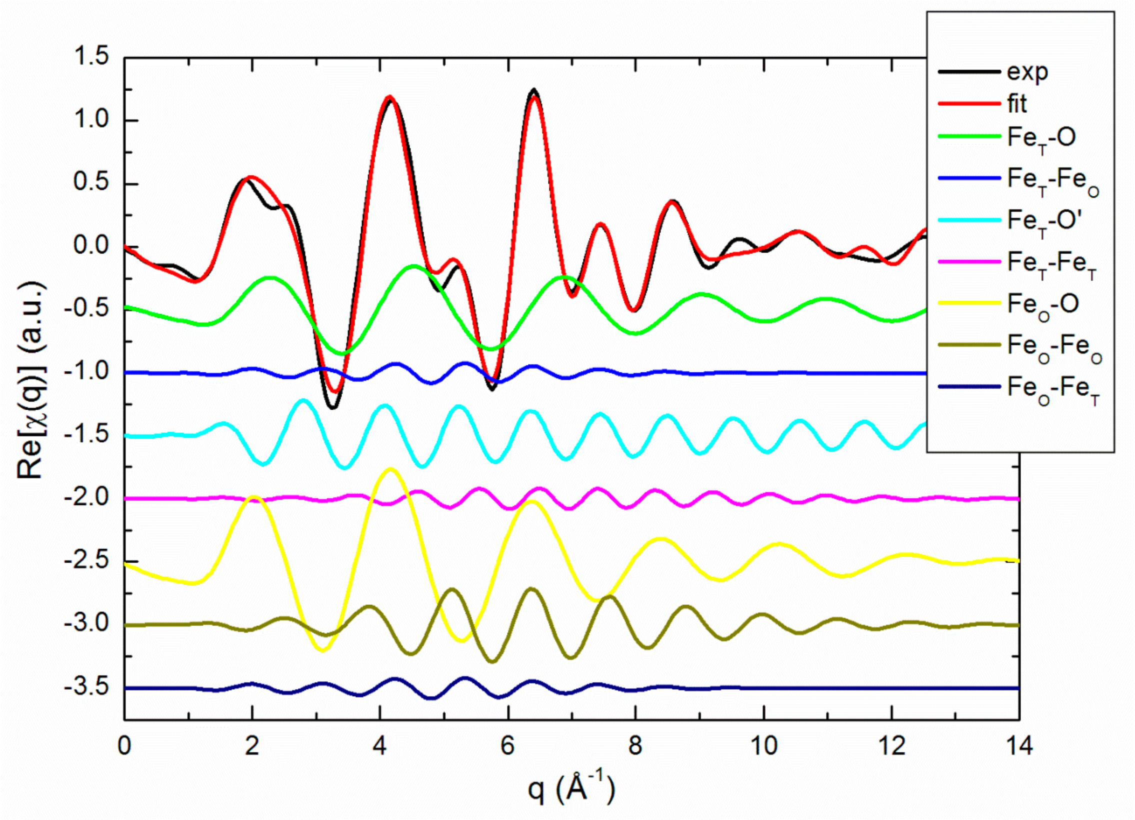

Figure S13b. Back Fourier transform best-fit curve and single contributions for sample P520.

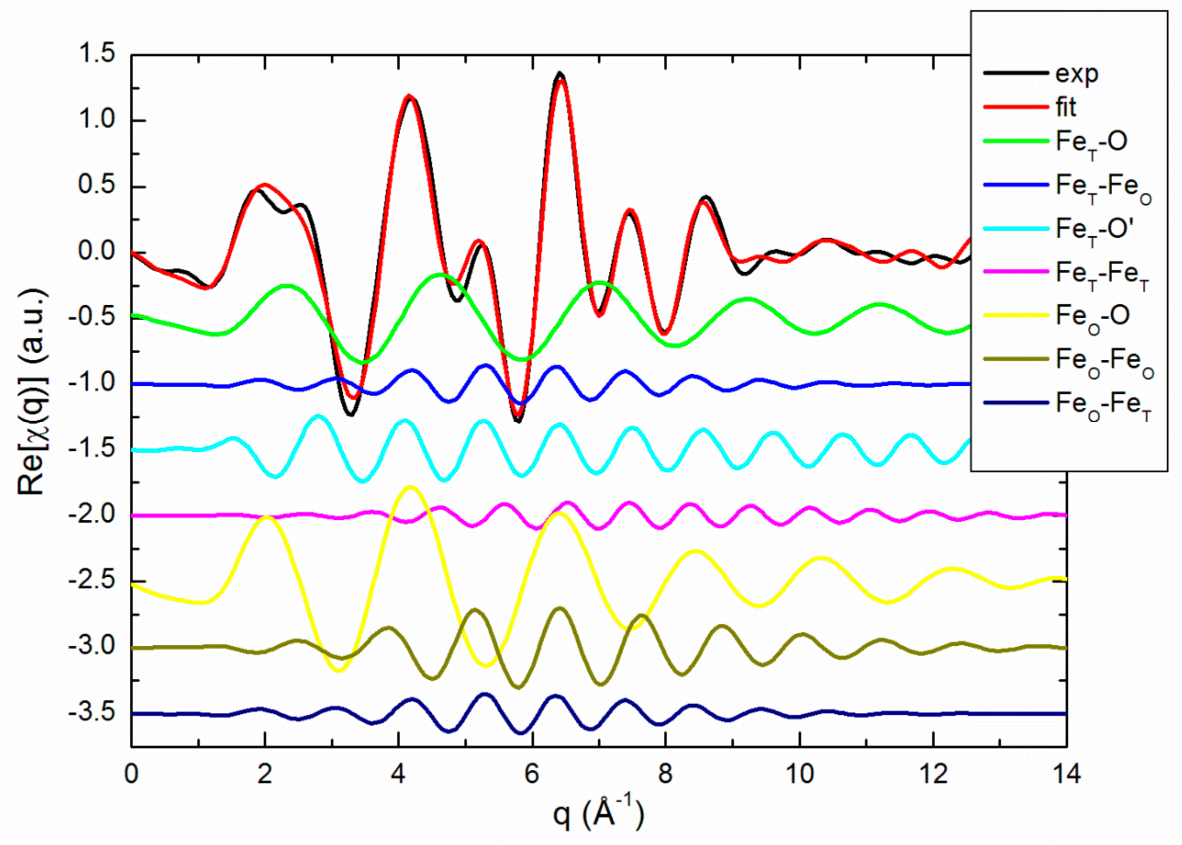

Figure S13c. Back Fourier transform best-fit curve and single contributions for sample Iolitec.

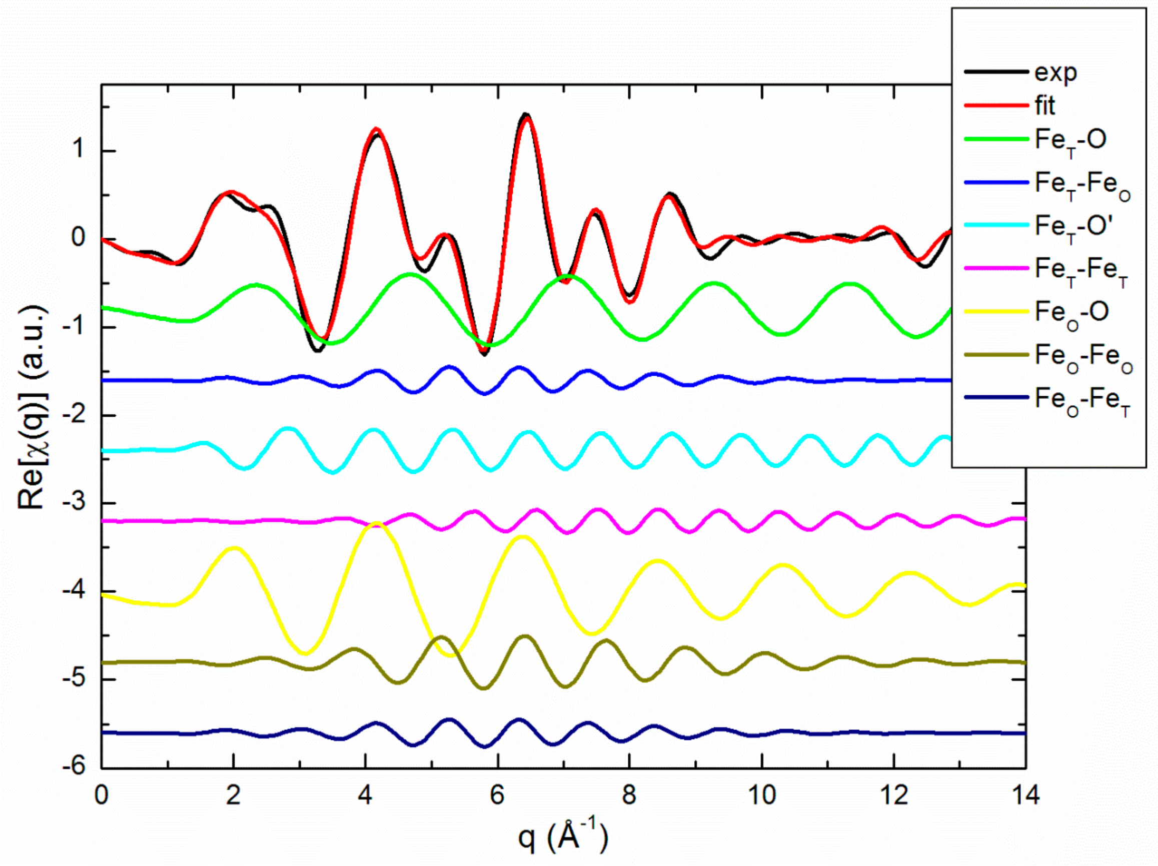

Figure S13d. Back Fourier transform best-fit curve and single contributions for sample Aldrich.

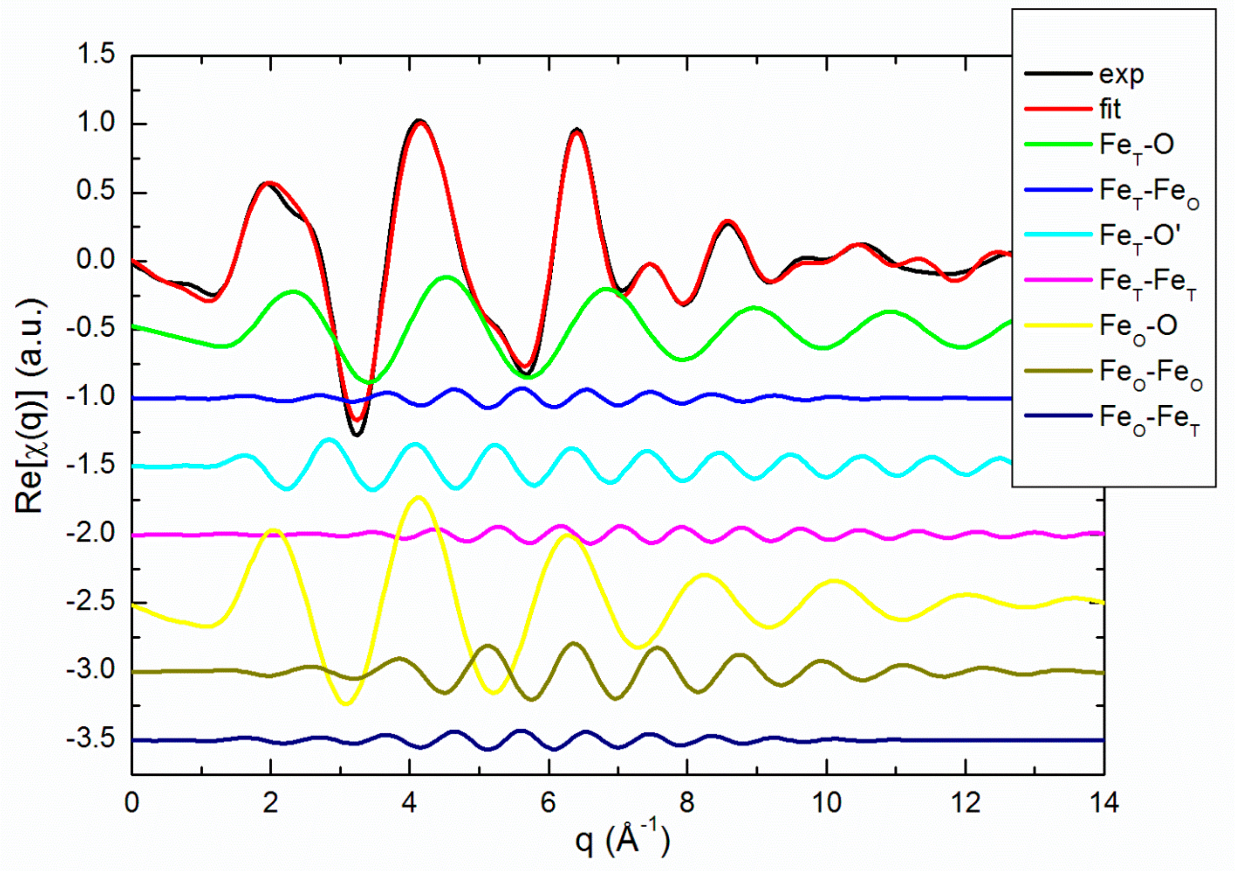

Figure S13e. Back Fourier transform best-fit curve and single contributions for sample P200 with the new coordination number CN′ values.

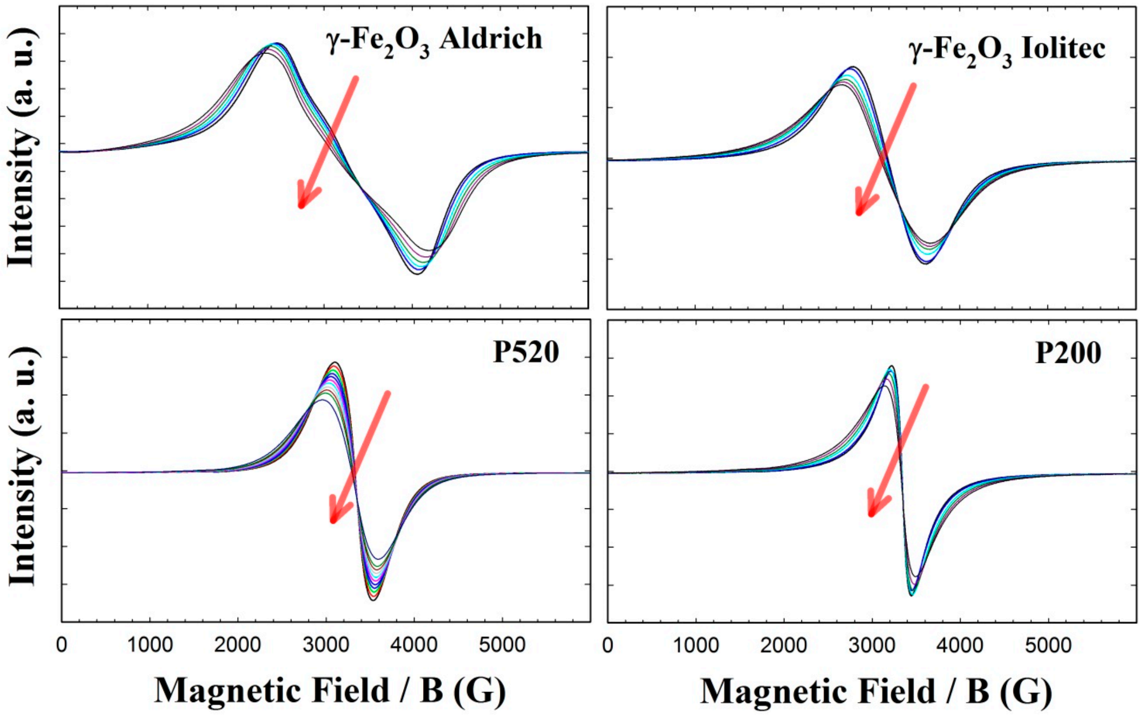

Figure S14. EMR spectra of γ-Fe_2O_3 samples heating up to 420 K. Red arrows indicate heating.
